# Supplementary material for: Predicting base editing outcomes with an attention-based deep learning algorithm trained on high-throughput target library screens
Source: Nat Commun. 2021 Aug 25;12:5114. doi: 10.1038/s41467-021-25375-z (PMC8387386; doi:10.1038/s41467-021-25375-z)
Supplement: Supplementary file 1 — Supplementary Information [file 41467_2021_25375_MOESM1_ESM.pdf]

Supplementary Information: Predicting base editing outcomes with an attention-based deep learning algorithm trained on target library screens

Kim F. Marquart[*1,2], Ahmed Allam[*3], Sharan Janjuha[*2], Anna Sintsova[§3], Lukas Villiger[†2], Nina Frey[1,2], Michael Krauthammer[#3], and Gerald Schwank[#2]

[1]Institute of Molecular Health Sciences, ETH Zurich, Zurich, Switzerland
[2]Department of Pharmacology and Toxicology, University of Zurich, Zurich, Switzerland
[3]Department of Quantitative Biomedicine, University of Zurich, Zurich, Switzerland

[§]Current address: Institute of Microbiology, ETH Zurich, Zurich, Switzerland
[†]Current address: McGovern Institute for Brain Research at MIT, Massachusetts Institute of Technology, Cambridge, MA, USA

[*]equal contribution
#corresponding

Supplementary Figures

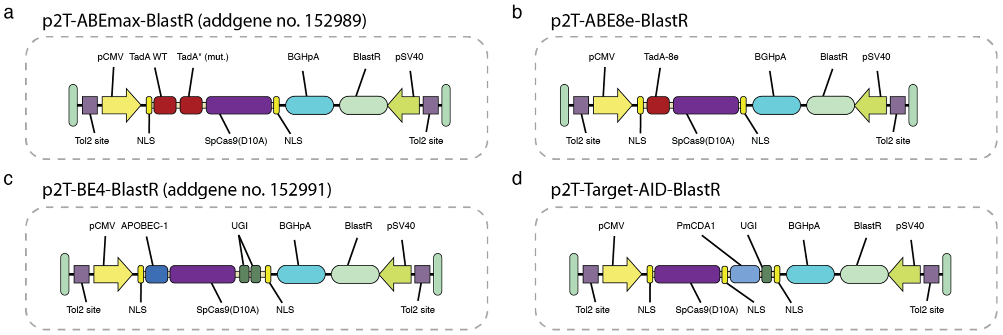

Supplementary Figure 1: **Plasmid maps of (a) ABEmax, (b) ABE8e, (c) CBE4max and (d) Target-AID.** pCMV, cytomegalovirus promoter; TadA WT, wild type Escherichia coli TadA (tRNA-specific adenosine deaminase) gene; TadA*, evolved Escherichia coli TadA variant gene (used in ABE7.10 variant); TadA-8e, evolved Escherichia coli TadA variant gene (used in ABE8e); APOBEC-1, apolipoprotein B mRNA editing enzyme catalytic subunit 1 gene; UGI, uracil DNA glycosylase inhibitor gene; PmCDA1, Petromyzon marinus cytidine deaminase 1 BlastR, blasticidin resistance gene; bGH, bovine growth hormone polyadenylation signal; Streptococcus pyogenes Cas9 with D10A (nickase) mutation; NLS, nuclear localization signal; pSV40, simian virus 40 promoter; Tol2 site, recognition element for Tol2 transposon.

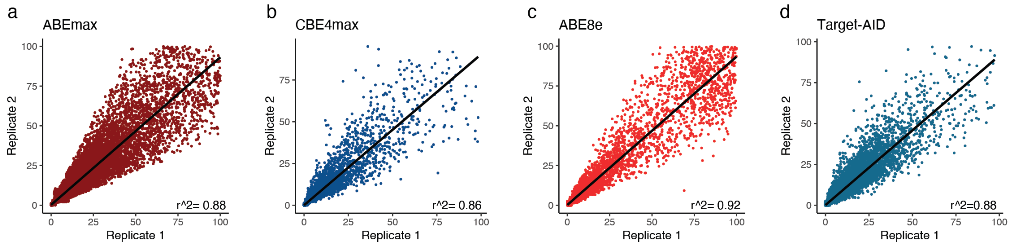

Supplementary Figure 2: **Correlations between the results of independent replicates of high-throughput base editor screening experiments.** Scatter plots show the correlation between editing efficiencies for all target bases that were edited in the two independent transfections of library-cells with **(a)** ABEmax, **(b)** CBE4max, **(c)** ABE8e and **(d)** Target-AID. Pearson's correlation coefficient (r^2) is shown. At 10 days post-transfection, the target sequences were PCR amplified and subjected to deep sequencing to measure editing at target bases.

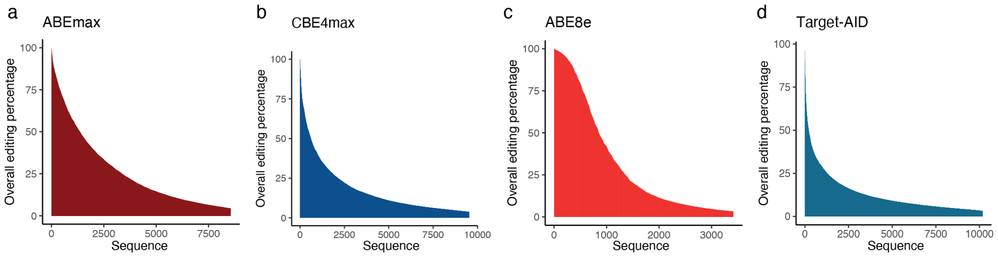

Supplementary Figure 3: **Editing rates in the self-targeting pooled library.** Percentage of overall editing efficiency scores above mean editing efficiency per target sequence for **(a)** ABEmax, **(b)** CBE4max, **(c)** ABE8e and **(d)** Target-AID.

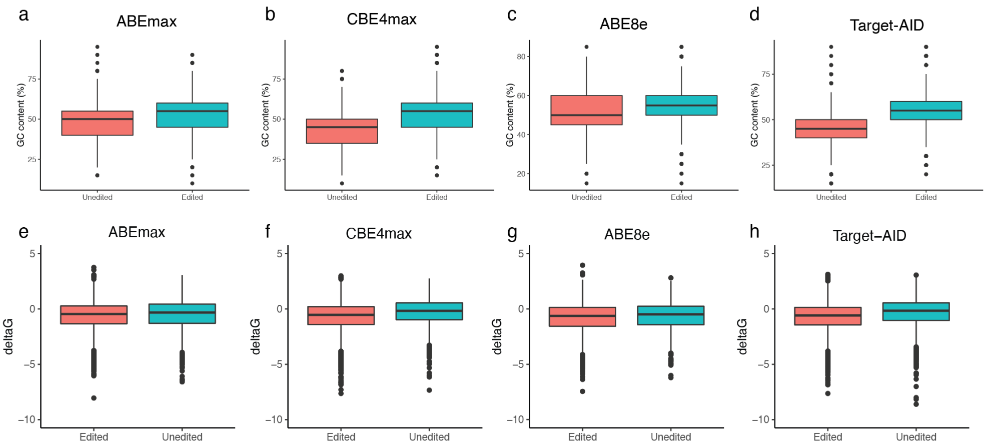

Supplementary Figure 4: **GC-content and Gibbs free energy for ABEmax and CBE4max in edited sequences and non-edited sequences**Boxplots show the distribution of GC content **(a-d)** and delta G values **(e-h)** of all edited and unedited target sequences for ABEmax, CBE4max, ABE8e and Target-AID. Box plots indicate median (middle line), 25th, 75th percentile (box) and 5th and 95th percentile (whiskers) as well as outliers (single points).

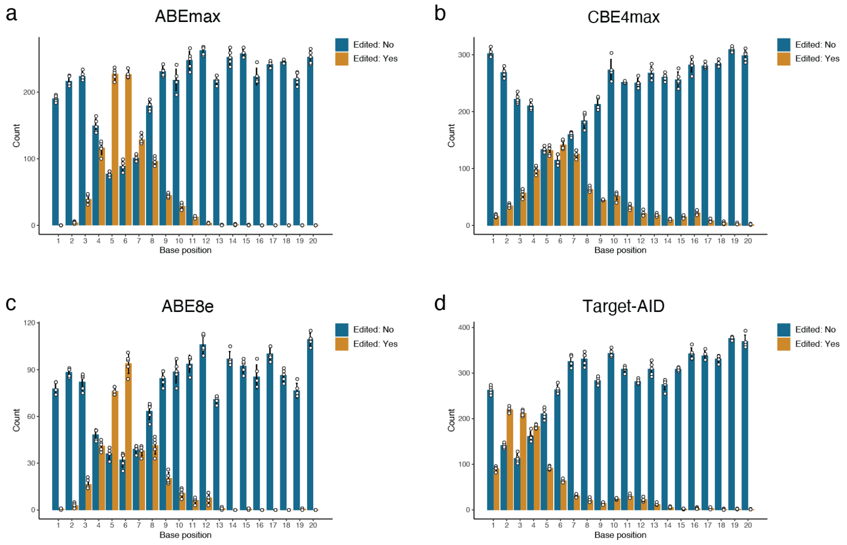

Supplementary Figure 5: **Per position base conversion (A-to-G; C-to-T) in the test data set used for the (a) ABEmax, (b) CBE4max, (c) ABE8e and (d) Target-AID model.** Bars represent mean of 5 model runs depicted as dots. Error bars represents standard deviation.

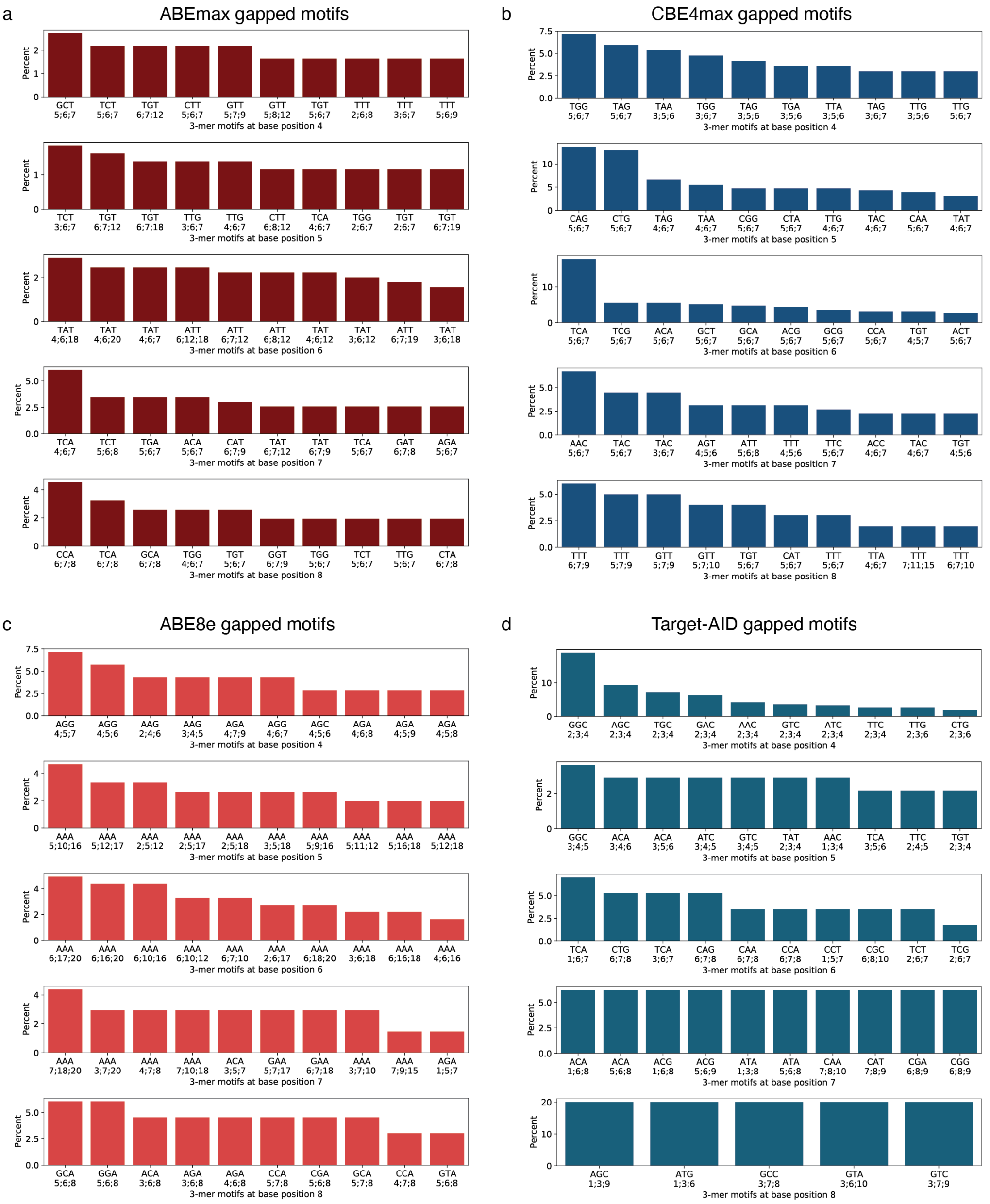

Supplementary Figure 6: Gapped 3-mer motifs for (a) ABEmax, (b) CBE4max, (c) ABE8e and (d) Target-AID. BE-DICT generates attention scores for every base in the 20-bp target region. Non-consecutive (gapped) attention score patterns for edited substrate nucleotides at positions within the editing window for each base editor are shown with the respective nucleotide positions.

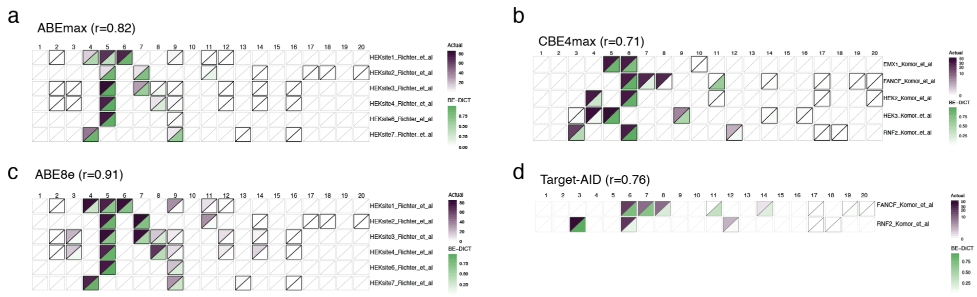

Supplementary Figure 7: Comparison of predicted (BE-DICT) versus published base editing activities on genomic loci. Heatmap shows the BE-DICT prediction values (green) and the editing percentage (purple) for target sequences treated with **(a)** ABEmax (Richter et al., 2020 [1]), **(b)** CBE4max (Komor et al., 2017 [2]), **(c)** ABE8e (Richter et al. 2020 [1]) and **(d)** Target-AID (Komor et al. 2017 [2]).

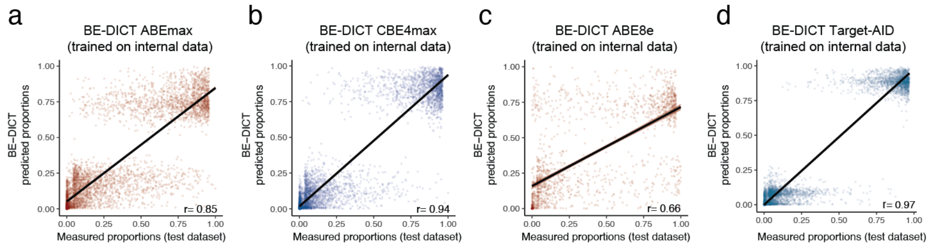

Supplementary Figure 8: **Predictions of the BE-DICT bystander module plotted against experimental results.(a-d)** BE-DICT bystander module trained and tested on datasets of our study. Pearson's correlation coefficient (r) is shown.

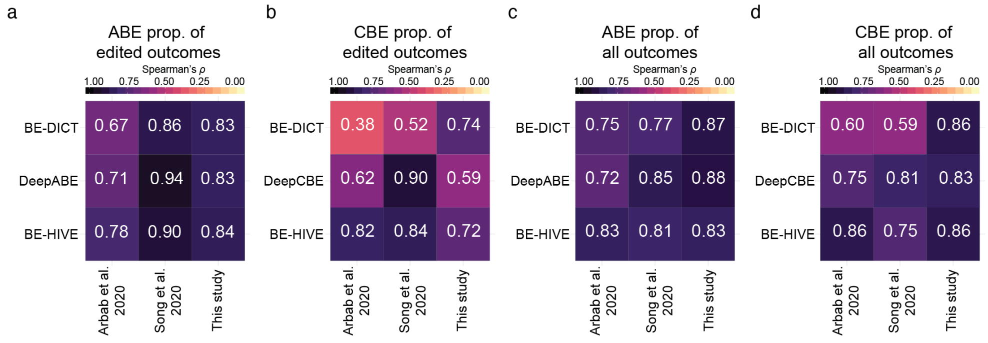

Supplementary Figure 9: **Performance evaluation of different machine learning models [3, 4] for ABE and CBE on prediction of the proportion of edited outcomes (a, b) and the prediction of all outcomes (c, d).** Spearmans's correlation (rho) was calculated by comparison of predicted versus measured base editing outcome proportions in datasets published by Arbab et al. [4] and Song et al. [3] and in this study. The number of analyzed outcomes in the dataset from Arbab et al. are n = 7743 (ABE edited outcomes), n = 7537 (CBE edited outcomes), n = 9008 (ABE all outcomes) and n = 8895 (CBE all outcomes) arising from a total of 1265 unique sequences for ABE and 1358 sequences for CBE. The number of analyzed outcomes in the dataset from Song et al. are n = 1767 (ABE edited outcomes), n = 2332 (CBE edited outcomes), n = 2204 (ABE all outcomes) and n = 2807 (CBE all outcomes) arising from a total of 437 unique sequences for ABE and 475 sequences for CBE. The number of analyzed outcomes in the dataset from this study are n = 3844 (ABE edited outcomes), n = 4502 (CBE edited outcomes), n = 5510 (ABE all outcomes) and n = 6176 (CBE all outcomes) arising from a total of 1667 unique sequences for ABE and 1675 sequences for CBE.

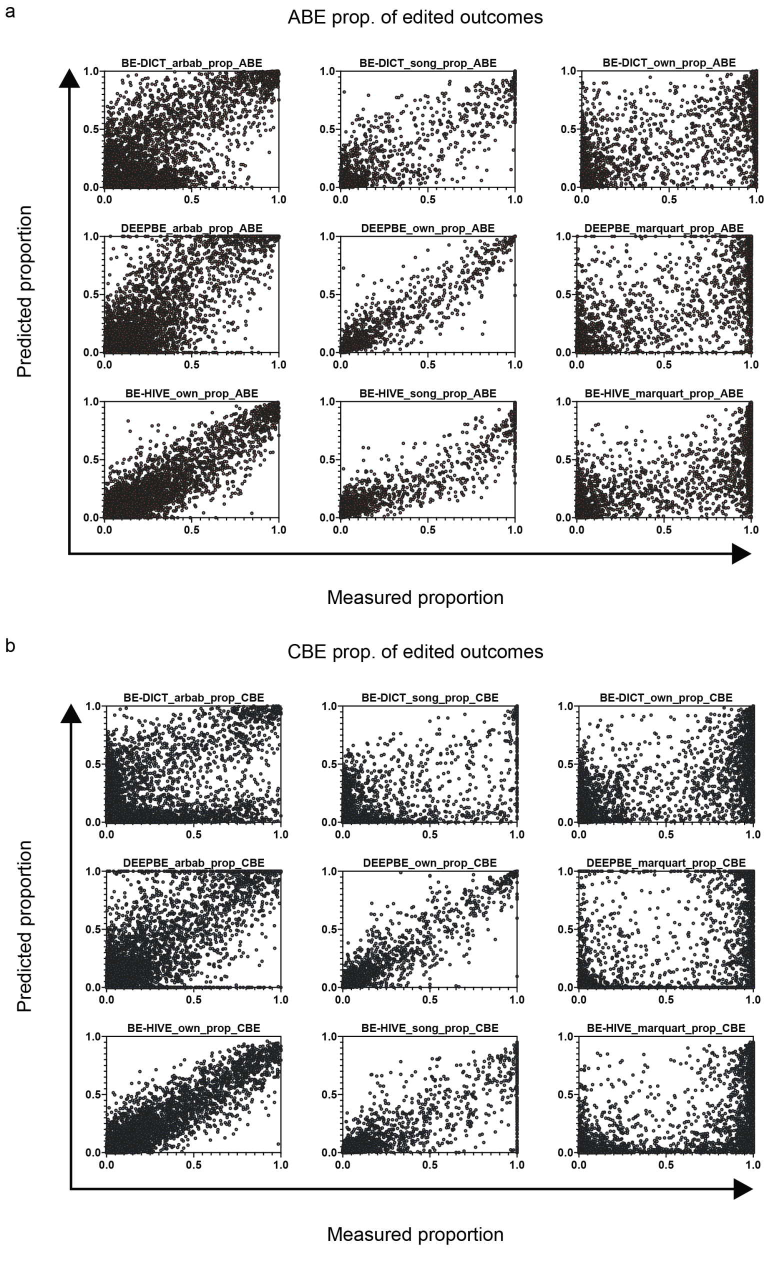

Supplementary Figure 10: Continued on next page.

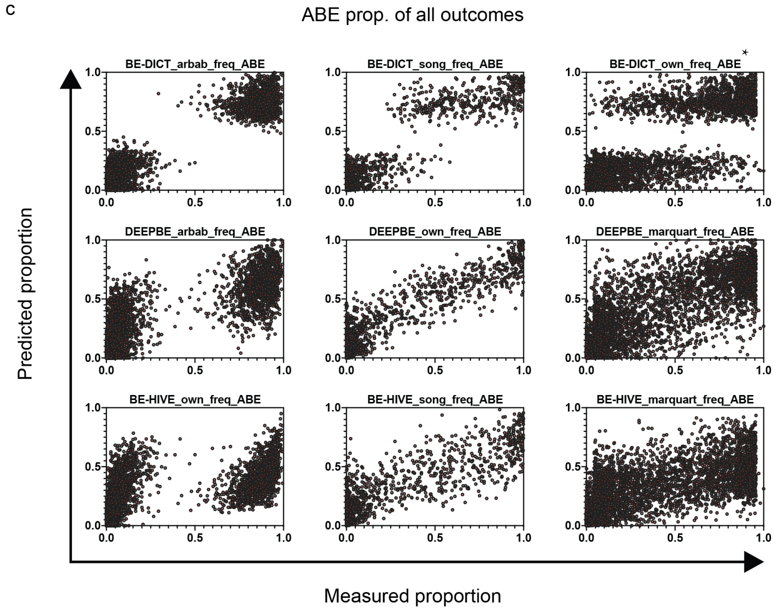

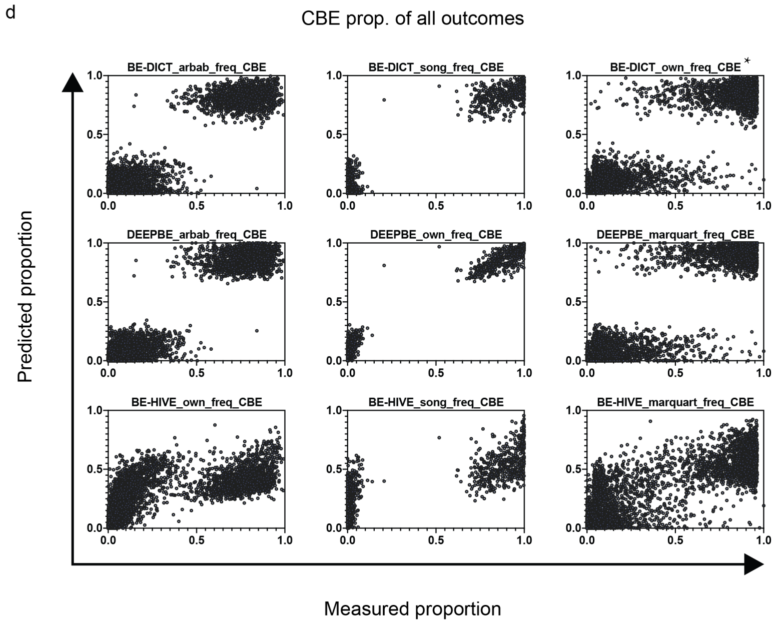

Supplementary Figure 10: **Performance evaluation of different machine learning models [3, 4] for predicting ABE and CBE editing outcomes.** Dot plots of data shown in Figure 5e-h. Plots show the correlation between prediction and ground truth of **(a, b)** edited outcomes and **(c, d)** all outcomes. The number of analyzed outcomes in the dataset from Arbab et al. are n = 7743 (ABE edited outcomes), n = 7537 (CBE edited outcomes), n = 9008 (ABE all outcomes) and n = 8895 (CBE all outcomes) arising from a total of 1265 unique sequences for ABE and 1358 sequences for CBE. The number of analyzed outcomes in the dataset from Song et al. are n = 1767 (ABE edited outcomes), n = 2332 (CBE edited outcomes), n = 2204 (ABE all outcomes) and n = 2807 (CBE all outcomes) arising from a total of 437 unique sequences for ABE and 475 sequences for CBE. The number of analyzed outcomes in the dataset from this study are n = 3844 (ABE edited outcomes), n = 4502 (CBE edited outcomes), n = 5510 (ABE all outcomes) and n = 6176 (CBE all outcomes) arising from a total of 1667 unique sequences for ABE and 1675 sequences for CBE. Negative predictions from DeepBaseEditor (DEEPBE) are not plotted. *The datasets 'BE-DICT_own_freq_ABE' and 'BE-DICT_own_freq_CBE' are also shown in Supplementary Fig. 8a, b and are included here to facilitate direct comparison to the other datasets.

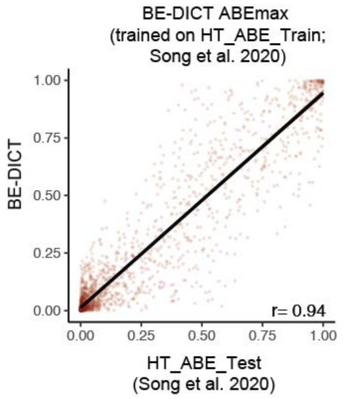

Supplementary Figure 11: **Predictions of the BE-DICT bystander module plotted against experimental results from Song et al. [3].** BE-DICT bystander module trained on the dataset provided by Song et al. (HT-ABE-Train), and tested on the (HT-ABE-Test) dataset of Song et al. [3]. Pearson's correlation coefficient (r) is shown.

Supplementary Notes

Supplementary Note 1: Attention-based Neural Network

1.1 Per-base Model Overview

We designed and implemented a multi-head self-attention model (named BE-DICT) inspired by the Transformer [5] encoder architecture. BE-DICT is implemented in PyTorch [6] and takes a sequence of nucleotides (i.e. using protospacer sequence of 20 bp window) as input and computes the probability of editing for each target nucleotide as output. The target nucleotides in our experiments were base *A* for ABEmax and ABE8e editors, and *C* for CBE4max and Target-AID base editors respectively.
The model has three main blocks: An (1) **Embedding block** that embeds both the nucleotide's and its corresponding position from one-hot encoded representation to a dense vector representation.
An (2) **Encoder block** that contains (a) a self-attention layer (with multi-head support), (b) layer normalization & residual connections (⇢), and (c) feed-forward network.
Lastly, an (3) **Output block** that contains (a) a position attention layer and (b) a classifier layer.
A formal description of each component of the model is described in their respective sections below.

1.2 Embedding Block

Formally, given a protospacer sequence $\underline{S} = [x_1, x_2, \cdots, x_T]$, a nucleotide at position t is represented by 1-of-*K* encoding where K is the size of the set of all nucleotide letters in the data such that $x_t \in [0,1]^K$. An embedding matrix W_e is used to map the input x_t to a fixed-length vector representation (Eq. 1)

$$e_t = W_e x_t \tag{1}$$

where $W_e \in \mathbb{R}^{d_e \times K}$, $e_t \in \mathbb{R}^{d_e}$, and d_e is the dimension of vector e_t.
Similarly, each nucleotide's position p_t in the sequence $\underline{S}$ is represented by 1-of-*T* encoding where T is the number of elements in the sequence (i.e. length of protospacer sequence) such that $p_t \in [0,1]^T$. An embedding matrix $W_{p'}$ is used to map the input p_t to a fixed-length vector representation (Eq. 2)

$$p'_t = W_{p'} p_t \tag{2}$$

where $W_{p'} \in \mathbb{R}^{d_{p'} \times T}$, $p'_t \in \mathbb{R}^{d_{p'}}$ and $d_{p'}$ is the dimension of vector p'_t such that d_e and $d_{p'}$ were equal (denoted by d from now on).
Both embeddings e_t and p'_t were summed (Eq. 3) to get a unified representation for every element in the sequence $\underline{S}$ (i.e. compute a new sequence $\underline{U} = [u_1, u_2, \cdots, u_T]$ where $u_t \in \mathbb{R}^d$, $\forall t \in [1, \cdots, T]$).

$$u_t = e_t + p'_t \tag{3}$$

1.3 Encoder Block

1.3.1 Self-Attention Layer

We followed a multi-head self-attention approach where multiple single-head self-attention layers are used in parallel (i.e. simultaneously) to process each input vector u_t. The outputs from every single-head layer are concatenated and transformed to generate a fixed-length vector using an affine transformation. The single-head self-attention approach [5] performs linear transformation to the input vector using three separate matrices: (1) a queries matrix W_{query}, (2) keys matrix W_{key}, and (3) values matrix W_{value}. Each input u_t in $\underline{U}$ is mapped using these matrices to compute three new vectors (Eq. 4, 5, and 6)

$$q_t = W_{query} u_t \tag{4}$$

$$k_t = W_{key} u_t \tag{5}$$

$$v_t = W_{value} u_t \tag{6}$$

where W_{query}, W_{key}, $W_{value} \in \mathbb{R}^{d' \times d}$, q_t, k_t, $v_t \in \mathbb{R}^{d'}$ are query, key and value vectors, and d' is the dimension of the three computed vectors respectively. In a second step, attention scores are computed using the pairwise

similarity between the query and key vectors for each position t in the sequence. The similarity is defined by computing a scaled dot-product between the pairwise vectors. At each position t, we compute attention scores α_{tl} representing the similarity between q_t and vectors k_l $\forall l \in [1, \ldots, T]$ (Eq. 7, 8) normalized using $softmax$ function. Then a weighted sum using the attention scores α_{tl} and value vectors v_l $\forall l \in [1, \ldots, T]$ is performed (Eq. 9) to generate a new vector representation $z_t \in \mathbb{R}^{d'}$ at position t. This process is applied to every position in the sequence $\underline{U}$ to obtain a sequence of vectors $\underline{Z} = [z_1, z_2, \cdots, z_T]$.

$$\alpha_{tl} = \frac{\exp\left(score(q_t, k_l)\right)}{\sum_{l=1}^{T} \exp\left(score(q_t, k_l)\right)} \tag{7}$$

$$score(q_t, k_l) = \frac{{q_t}^{\top} k_l}{\sqrt{d}} \tag{8}$$

$$z_t = \sum_{l=1}^{T} \alpha_{tl} v_l \tag{9}$$

In a multi-head setting with H number of heads, the queries, keys and values matrices will be indexed by superscript h (i.e. W^h_{query}, W^h_{key}, $W^h_{value} \in \mathbb{R}^{d' \times d}$) and applied separately to generate a new vector representation z^h_t for every single-head self-attention layer. The output from each single-head layer is concatenated into one vector $z^{concat}_t = concat(z^1_t, z^2_t, \cdots, z^H_t)$ where $z^{concat}_t \in \mathbb{R}^{d'H}$ and then transformed using affine transformation (Eq. 10) such that $W_{unify} \in \mathbb{R}^{d' \times d'H}$ and $b_{unify} \in \mathbb{R}^{d'}$. This process is applied to each position in the sequence $\underline{Z}$ to generate a sequence $\underline{\tilde{Z}} = [\tilde{z}_1, \tilde{z}_2, \cdots, \tilde{z}_T]$.

$$\tilde{z}_t = W_{unify} z^{concat}_t + b_{unify} \tag{10}$$

1.3.2 Layer Normalization & Residual Connections

We used residual connections / skip-connections (⇢) [7] in order to improve the gradient flow in layers during training. This is done by summing both the newly computed output of the current layer with the output from the previous layer. In our setting, a first residual connection sums the output of the self-attention layer $\tilde{z}_t$ and the output of embedding block u_t for each position t in the sequence. We will refer to the summed output by $\tilde{z}_t$ for simplicity.

Layer normalization [8] was used in two occasions; after the self-attention layer and feed-forward network layer with the goal to ameliorate the "covariate-shift" problem by re-standardizing the computed vector representations (i.e. using the mean and variance across the features/embedding dimension d'). Given a computed vector $\tilde{z}_t$, $LayerNorm$ function will standardize the input vector using the mean μ_t and variance σ^2_t along the features dimension d' and apply a scaling γ and shifting step β (Eq. 13). γ and β are learnable parameters and ϵ is small number added for numerical stability.

$$\mu_t = \frac{1}{d'} \sum_{j=1}^{d'} \tilde{z}_{tj} \tag{11}$$

$$\sigma^2_t = \frac{1}{d'} \sum_{j=1}^{d'} (\tilde{z}_{tj} - \mu_t)^2 \tag{12}$$

$$LayerNorm(\tilde{z}_t) = \gamma \times \frac{\tilde{z}_t - \mu_t}{\sqrt{\sigma^2_t + \epsilon}} + \beta \tag{13}$$

1.3.3 FeedForward Layer

After a layer normalization step, a feed-forward network consisting of two affine transformation matrices and non-linear activation function is used to further compute/embed the learned vector representations from previous layers. The first transformation (Eq. 14) uses $W_{MLP1} \in \mathbb{R}^{\xi d' \times d'}$ and $b_{MLP1} \in \mathbb{R}^{\xi d'}$ to transform input $\tilde{z}_t$ to new vector $\in \mathbb{R}^{\xi d'}$ where $\xi \in \mathbb{N}$ is multiplicative factor. A non-linear function such as $ReLU(z) = max(0, z)$ is applied followed by another affine transformation using $W_{MLP2} \in \mathbb{R}^{d' \times \xi d'}$ and $b_{MLP2} \in R^{d'}$ to obtain vector $r_t \in \mathbb{R}^{d'}$. A layer normalization (Eq. 15) is applied to obtain $\tilde{r}_t \in \mathbb{R}^{d'}$.

$$r_t = W_{MLP2} ReLU(W_{MLP1} \tilde{z}_t + b_{MLP1}) + b_{MLP2} \tag{14}$$

$$\tilde{r}_t = LayerNorm(r_t) \tag{15}$$

These transformations are applied to each vector in sequence $\underline{\tilde{Z}}$ to obtain new sequence $\underline{R} = [\tilde{r}_1, \tilde{r}_2, \cdots, \tilde{r}_T]$. At this point, the *encoder* block operations are done and multiple encoder blocks can be stacked in series for E number of times. In our experiments, E was a hyperparameter that was empirically determined using a validation set (as the case of the number of attention heads H used in self-attention layer).

1.4 Output Block

1.4.1 Position Attention Layer

The position attention layer is parametrized by a set of *global* context vectors $\underline{C} = [c_1, c_2, \cdots, c_T]$ corresponding to each position in the protospacer sequence. These context vectors are learnable parameters optimized during the training. For a target base at position t, attention scores $\psi_t \forall t \in [1, \cdots, T]$ are calculated using the pairwise similarity between the context vector $c_t \in \mathbb{R}^{d'}$ with the vectors representing every position in the protospacer sequence ($[\tilde{r}_1, \tilde{r}_2, \cdots, \tilde{r}_T]$ computed in the previous layer (Eq. 16, 17). These scores are normalized and used to compute weighted sum of the $[\tilde{r}_1, \tilde{r}_2, \cdots, \tilde{r}_T]$ vectors to generate a new vector representation $o_t \in \mathbb{R}^{d'}$ at position t. This process is done at each target position to generate a sequence $\underline{O} = [o_1, o_2, \cdots, o_T]$ that is further passed to the classifier layer.

$$\psi_t = \frac{\exp\left(score(c_t, \tilde{r}_t)\right)}{\sum_{j=1}^{T} \exp\left(score(c_t, \tilde{r}_j)\right)} \tag{16}$$

$$score(c_t, \tilde{r}_t) = \frac{{c_t}^\top \tilde{r}_t}{\sqrt{d'}} \tag{17}$$

$$o_t = \sum_{t=1}^{T} \psi_t \tilde{r}_t \tag{18}$$

1.4.2 Output Classifier

The last layer in the model takes as input the computed representation vectors $\underline{O} = [o_1, o_2, \cdots, o_T]$ from the *position attention* layer and performs an affine transformation followed by *softmax* operation to compute a probability distribution on the outcomes (i.e. edit vs. no edit) for every target base under consideration (i.e. base *A* or *C*). That is, the probability distribution $\hat{y}_t$ at position t is computed using Eq. 19

$$\hat{y}_t = \sigma(W_o o_t + b_o) \tag{19}$$

where $W_o \in \mathbb{R}^{|V_{outcome}| \times d'}$, $b_o \in \mathbb{R}^{|V_{outcome}|}$, $V_{outcome} \in \{0, 1\}$ is the set of admissible labels (binary variable in our case). Moreover, $|V_{outcome}|$ is the number of labels, d' is the dimension of o_t and σ is the $softmax$ function.

1.5 Objective Function

We defined the loss for an i-th sequence at each position t by the cross-entropy loss

$$l_t^{(i)} = -\sum_{c=1}^{|V_{outcome}|} y_{t,c}^{(i)} \times log(\hat{y}_{t,c}^{(i)}) \tag{20}$$

where the loss for the i-th sequence is defined by the average loss over the sequence length T

$$L_i = \frac{1}{T} \sum_{t=1}^{T} l_t^{(i)} \tag{21}$$

Given that our focus is on target nucleotides (i.e. bases *A* or *C* depending on the base editor used), our model's focus should be on positions where target bases occur. Hence, we modify the defined loss over i-th sequence (see Eq. 21) by defining an average loss for target base L_i^{target} (Eq. 22)

$$L_i^{target} = \frac{1}{\sum_{t=1}^{T} \mathbb{1}[x_{t,base}^{(i)} = 1]} \sum_{t=1}^{T} l_t^{(i)} \mathbb{1}[x_{t,base}^{(i)} = 1] \tag{22}$$

where $\mathbb{1}[x^{(i)}_{t,base} = 1]$ is an indicator function that is equal to 1 when x^i_t representing the nucleotide at position t for the i-th sequence is the target base (i.e. *A* for ABEmax and ABE8e and *C* for CBE4max and Target-AID base editors respectively). Lastly, the objective function for the whole training set D_{train} is defined by the average loss across all the sequences in D_{train} plus a weight regularization term (i.e. l_2-norm regularization) applied to the model parameters represented by $\boldsymbol{\theta}$

$$L(\boldsymbol{\theta}) = \frac{1}{N}\sum_{i=1}^{N} L_i^{target} + \frac{\lambda}{2}||\boldsymbol{\theta}||_2^2 \tag{23}$$

In practice, the training occurs using mini-batches where computing the loss function and updating the parameters/weight occur after processing each mini-batch of the training set.

Supplementary Note 2: Bystander Model

We next developed a *bystander* variation of the BE-DICT model, which is capable of predicting the relative proportions of different editing outcomes (combinations of target base and bystander conversions) per target locus (BE-DICT bystander module). The model is based on an encoder-decoder architecture (adapting the Transformer architecture used in the BE-DICT per-base model), which takes a sequence of nucleotides of the protospacer as input, and computes probabilities for all combinations of sequences with target-base and bystander transitions, as well as the probability of observing a wild-type sequence. The model uses similar **Embedding** and **Encoder** blocks (see sections 1.2 and 1.3 in the per-base model) for processing the input sequence (i.e. protospacer). Additionally, the model has a **Decoder** block that is used to compute the probability of the different editing outcome sequences (i.e. relative proportion of combinations of target base and bystander transitions).

2.1 Embedding & Encoder Block

Similar to the per-base model setting, the bystander model takes as input a protospacer sequence $\underline{S} = [x_1, x_2, \cdots, x_T]$, where a nucleotide at position t is represented by 1-of-*K* encoding and K is the size of the set of all nucleotide letters in the data such that $x_t \in [0,1]^K$. The sequence is passed to **Embedding** and **Encoder** blocks (see sections 1.2 and 1.3) to generate a new sequence of vectors denoted by $\underline{Z} = [\tilde{z}_1, \tilde{z}_2, \cdots, \tilde{z}_T]$ as an output. As before, multiple encoder blocks can be stacked and applied in series to generate the new sequence representation $\underline{Z}$.

2.2 Embedding & Decoder Block

The list of observed experimental edit combinations of the target nucleotides for every protospacer sequence $\underline{S}$ is used as input to the **Decoder** block. The edit combinations included canonical conversions corresponding to the chosen base editor (i.e. A->G conversions for ABEmax and ABE8e editors, and C->T conversions for CBE4max and Target-AID editors). A j-th output sequence is denoted by $\underline{O_j} = [o_{j1}, o_{j2}, \cdots, o_{jT}]$ where j indexes the list of observed edit combination sequences for the given input protospacer sequence.

Every output sequence $\underline{O_j}$ is passed to an **Embedding block** that embeds both the nucleotides and their corresponding position from one-hot encoded representation to a dense vector representation obtaining a new sequence $\underline{B_j}$ (similar to section 1.2).

Subsequently, a masked self-attention layer is used as an "autoregressive layer" to ensure the use of past information only while computing the new vector representation at each step in the sequence $\underline{E_j} = [e_{j1}, e_{j2}, \cdots, e_{jT}]$. Layer normalization and residual connections are used (similar to section 1.3.2 above) to obtain a sequence of vectors $\underline{V_j}$.

The sequence $\underline{V_j}$ is later passed to a cross-attention layer such that the vectors in $\underline{V_j}$ were used as *queries*, and the vectors in $\underline{Z}$ (the computed sequence from the **Encoder block**) as *keys* to compute the attention scores (i.e. pairwise similarity using scaled dot-product) between the query and key vectors. These scores are used to weigh the vectors in $\underline{V_j}$ when computing the new sequence representation $\underline{D_j} = [d_{j1}, d_{j2}, \cdots, d_{jT}]$.

Then a series of layer normalization, residual connections followed by feed-forward network are used to compute the penultimate sequence representation $\underline{H_j} = [h_{j1}, h_{j2}, \cdots, h_{jT}]$ (i.e. before passing it to the **output**

block). Multiple decoder blocks can be stacked and applied in series to generate the new sequence representation $\underline{H_j}$.

To summarize, the **Decoder block** consists of the following layers in this order: *Masked Self-Attention*, *Add+Normalize*, *Cross-Attention*, *Add+Normalize*, *Feed-Forward*, *Add+Normalize*.

2.3 Output block

The last layer in the model takes as input the computed representation $\underline{H_j} = [h_{j1}, h_{j2}, \cdots, h_{jT}]$ (i.e. sequence of vectors) from the **Decoder block** and performs an affine transformation followed by *softmax* operation to compute the probability of conversion for every target base (i.e. base *A* or *C* depending on the chosen base editor) in the j-th outcome sequence under consideration (Eq. 24)

$$\hat{y}_{jt} = \sigma(W h_{jt} + b_t) \tag{24}$$

where $W \in \mathbb{R}^{2\times d'}$, $b_t \in \mathbb{R}^2$, $h_{jt} \in \mathbb{R}^{d'}$ and σ is the $softmax$ function.
The probability of the whole outcome sequence $\hat{y}_j$ (i.e. $j-$th edit combination) is computed using Eq. 25

$$\hat{y}_j = \prod_{t=1}^{T} \hat{y}_{jt} \tag{25}$$

2.4 Objective Function

Kullback–Leibler divergence (KL-div) was used as a loss function for an i-th input sequence (i.e. protospacer). KL-div represents the relative entropy of the model's estimated distribution over all outcome sequences $\hat{y}_j$ with respect to the true distribution (i.e. observed proportions of editing outcomes) y_j for $j \in [1, \cdots, J]$ and J representing the number of edited outcome sequences for a given protospacer sequence (Eq. 26).

$$D^i_{KL-div}(y^i \| \hat{y}^i) = \sum_{j=1}^{J} y^i_j log(\frac{y^i_j}{\hat{y}^i_j}) \tag{26}$$

Lastly, the objective function for the whole training set is defined by the average loss across all the input protospacer sequences plus a weight regularization term (i.e. l_2-norm regularization) applied to the model parameters represented by $\boldsymbol{\theta}$

$$L(\boldsymbol{\theta}) = \frac{1}{N} \sum_{i=1}^{N} D^i_{KL-div}(y^i \| \hat{y}^i) + \frac{\lambda}{2} \|\boldsymbol{\theta}\|^2_2 \tag{27}$$

In practice, the training occurs using mini-batches where computing the loss function and updating the parameters/weight occur after processing each mini-batch of the training set.

Supplementary Note 3: Experiments for machine learning

3.1 Training & Evaluation Workflow

For model training (per-base and bystander) we used ≈ 80% of the dataset consisting of randomized-DNA target sequences, and performed stratified random splits for the rest of the sequences to generate an equal ratio (1:1) between test and validation datasets. We repeated this process five times (denoted by runs), in which we trained and evaluated a model for each base editor separately for each run.

3.1.1 Per-base Model

For the per-base model, training examples were weighted inversely proportional to class/outcome frequencies in the training data. BE-DICT performance was evaluated using area under the receiver operating characteristic curve (AUC), and area under the precision recall curve (AUPR). During training of the models, the epoch in which the model achieved the best AUPR on the validation set was recorded, and model state as it was trained up to that epoch was saved. This best model, as determined by the validation set, was then tested on the test

split. The evaluation of the trained models for each base editor was based on their average performance on the test sets across the five runs.
We further compared BE-DICT's per position accuracy with a majority class predictor (one for each base editor) that uses the training data to estimate the per position prior (i.e. base rate) probability of having a target base edited. Based on the estimated prior, the predictor assigns the majority class (one with higher probability) as the outcome for the target base at the position under consideration.

3.1.2 Bystander Model

The bystander model performance was evaluated using Pearson and Spearman correlation. During training of the models, the epoch in which the model achieved the best harmonic mean between both scores on the validation set was recorded, and model state as it was trained up to that epoch was saved. This best model, as determined by the validation set, was then tested on the test split. The evaluation of the trained models for each base editor was based on their average performance on the test sets across the five runs.

3.2 Hyperparameters Optimization

We used a uniform random search strategy [9] that randomly chose a set of hyperparameters configurations (i.e. embedding dimension, number of attention heads, dropout probability, etc.) from the set of all possible configurations. Then the best configuration for each model (i.e. the one achieving best performance on the validation set) was used for the final training and testing.
The range of possible hyperparameters configuration (i.e. choice of values for hyperparameters) for BE-DICT models is reported in Tables 1 and 2 for per-base and bystander model respectively. In our experiments, there was an overlap between the best hyperparameter configurations for the different models trained on the various base editors. Hence, we opted for a common hyperparameter configuration among the trained models.

List 1 BE-DICT (per-base) hyperparameters options

- Embedding Block operations
  - Nucleotide embedding layer
    - embedding dimension d' .... $\{16, 32, 64, 128\}$
  - Position embedding layer
    - embedding dimension d' .... $\{16, 32, 64, 128\}$
- Encoder Block operations
  - Self-attention layer
    - Number of attention heads H' .... $\{2, 4, 6, 8, 12\}$
    - Attention type .... $\{Wide\}$
  - Dropout .... $\{0.1, 0.3, 0.5\}$
  - Feed-Forward layer
    - MLP embedding factor (multiplier) ξ .... $\{2, 3\}$
    - Non-linear function .... $\{tanh, ELU, ReLU\}$
  - Number of repeats for Encoder Block .... $\{1, 2, 4, 6\}$
- l_2-norm regularization λ .... $\{10^{-5}, 10^{-4}, 10^{-3}, 10^{-2}, 10^{-1}\}$
- Batch size during training $|B|$ .... $\{500, 1000, 2000, 3000, 4000\}$
- Optimization algorithm .... $\{Adam\}$

List 2 BE-DICT (bystander) hyperparameters options

- Embedding Block operations
  - Nucleotide embedding layer
    - embedding dimension d' .... $\{16, 32, 64, 128\}$
  - Position embedding layer
    - embedding dimension d' .... $\{16, 32, 64, 128\}$
- Encoder Block operations
  - Self-attention layer
    - Number of attention heads .... $\{2, 4, 6, 8, 12\}$
    - Attention type .... $\{Wide, Narrow\}$
  - Dropout .... $\{0.1, 0.25, 0.45\}$
  - Feed-Forward layer
    - MLP embedding factor (multiplier) ξ .... $\{2, 3\}$
    - Non-linear function .... $\{tanh, ELU, ReLU\}$
  - Number of repeats for Encoder Block .... $\{1, 2, 4\}$
- Decoder Block operations
  - Masked Self-attention layer
    - Number of attention heads .... $\{2, 4, 6, 8, 12\}$
    - Attention type .... $\{Wide, Narrow\}$
  - Cross-attention layer
    - Number of attention heads .... $\{2, 4, 6, 8, 12\}$
    - Attention type .... $\{Wide, Narrow\}$
  - Dropout .... $\{0.1, 0.25, 0.45\}$
  - Feed-Forward layer
    - MLP embedding factor (multiplier) ξ .... $\{2, 3\}$
    - Non-linear function .... $\{tanh, ELU, ReLU\}$
  - Number of repeats for Decoder Block .... $\{1, 2, 4\}$
- l_2-norm regularization λ .... $\{10^{-5}, 10^{-4}, 10^{-3}, 10^{-2}, 10^{-1}\}$
- Batch size during training $|B|$ .... $\{300, 400, 500, 1000, 1500, 2000\}$
- Optimization algorithm .... $\{Adam\}$

Supplementary Note 4:
DNA used in this study

4.1 Oligonucleotide pool design

Oligonucleotide sequences, each containing a guide RNA and corresponding target sequence pair, used to generate the pooled library.

Primer binding sites
Guide RNA sequence
Spcas9 guide RNA scaffold
Target sequence with PAM
Randomized barcode 1 and randomized barcode 2

ATCTTGTGGAAAGGACGAAACACCGNNNNNNNNNNNNNNNNNNNGTTTTAGA
GCTAGAAATAGCAAGTTAAAATAAGGCTAGTCCGTTATCAACTTGAAAAAGTGG
CACCGAGTCGGTGCTTTTTTNNNNNNNNNNNNNNNNNNNNNNNNNNNNNNNGGNNN
NNNGCTCTACCACTTGTACTTCAGC

4.2 Primers used for high-throughput sequencing

Primer used for:		Sequence (5'-3')
Oligo-pool amplification	FW	ATCTTGGGAAAGGACGAAACACC
	RV	GCTGAAGTACAAGTGGTAGAGC
Pooled screens (PCR on genomic DNA)	FW	CTTTCCCTACACGACGCTCTTCCGATCTNNNNNNNNGAAAAAGTGGCACCGAGTCG
	RV	GGAGTTCAGACGTGTGCTCTTCCGATCTNNNNNNNNACTATTCTTTCCCCTGCACTGT
DOCK3 no2	FW	CTTTCCCTACACGACGCTCTTCCGATCTNNNNTCCTACATTTCAGTGAGCGGT
	RV	GGAGTTCAGACGTGTGCTCTTCCGATCTNNNNGCAGGCCACTGGTTAGAGTC
TARDBP no2	FW	CTTTCCCTACACGACGCTCTTCCGATCTNNNNGCGCTGTACAGAGGACATGA
	RV	GGAGTTCAGACGTGTGCTCTTCCGATCTNNNNCCTGAATGGCTTGGGGATGA
ZNF212 no1	FW	CTTTCCCTACACGACGCTCTTCCGATCTNNNNGCCTGCACAGTGAGGAAGAG
	RV	GGAGTTCAGACGTGTGCTCTTCCGATCTNNNNCCAGAGCCTGTTGGAAGCC

NEK1 no2	FW	CTTTCCCTACACGACGCTCTTCCGATCTNNNNAGCCATGCTTTTGATGTACGT
	RV	GGAGTTCAGACGTGTGCTCTTCCGATCTNNNNAACAGATCCCCTCCCTCACA
RSF1 no1	FW	CTTTCCCTACACGACGCTCTTCCGATCTNNNNCCCCTTCTCTCCTCCCCTTC
	RV	GGAGTTCAGACGTGTGCTCTTCCGATCTNNNNTTGTTCTCGAGAAGTCCCGC
RSF1 no2	FW	CTTTCCCTACACGACGCTCTTCCGATCTNNNNAGCTAGAAAAACCTTTGCCAGA
	RV	GGAGTTCAGACGTGTGCTCTTCCGATCTNNNNGGCTTCTCCATGCTACTTTTGG
FANCF no3	FW	CTTTCCCTACACGACGCTCTTCCGATCTNNNNCTACCTGCGCCACATCCATC
	RV	GGAGTTCAGACGTGTGCTCTTCCGATCTNNNNTTCGCTAATCCCGGAACTGG
FANCF no5	FW	CTTTCCCTACACGACGCTCTTCCGATCTNNNNCTACCTACGTCAGCACCTGG
	RV	GGAGTTCAGACGTGTGCTCTTCCGATCTNNNNGATGGATGTGGCGCAGGTAG
EMX1 no1	FW	CTTTCCCTACACGACGCTCTTCCGATCTNNNNCAGGTGAAGGTGTGGTTCCA
	RV	GGAGTTCAGACGTGTGCTCTTCCGATCTNNNNCACCGGTTGATGTGATGGGA
EMX1 no2	FW	CTTTCCCTACACGACGCTCTTCCGATCTNNNNATAGTCCCCTTGGGGTGACA
	RV	GGAGTTCAGACGTGTGCTCTTCCGATCTNNNNCCGGCCAGAGACTTCCTGTA
HEK site no1	FW	CTTTCCCTACACGACGCTCTTCCGATCTNNNNCCAGCCCCATCTGTCAAACT
	RV	GGAGTTCAGACGTGTGCTCTTCCGATCTNNNNTGAATGGATTCCTTGGAAACAATGA
HEK site no2	FW	CTTTCCCTACACGACGCTCTTCCGATCTNNNNAGAGACTGATTGCGTGGAGT
	RV	GGAGTTCAGACGTGTGCTCTTCCGATCTNNNNCACTCCAGCCTAGGCAACAA
HEK site no3	FW	CTTTCCCTACACGACGCTCTTCCGATCTNNNNCCGACAGCCAGTGGTTAAGT
	RV	GGAGTTCAGACGTGTGCTCTTCCGATCTNNNNGCTTTTCACCGACTGCACAG
HEK site no8	FW	CTTTCCCTACACGACGCTCTTCCGATCTNNNNCCCTGTTCCTAAAGCCCACC
	RV	GGAGTTCAGACGTGTGCTCTTCCGATCTNNNNACTGGTTCTGTTTGTGGCCA
HEK site no9	FW	CTTTCCCTACACGACGCTCTTCCGATCTNNNNTTGCTTATTGCTGAGGGGCA

	RV	GGAGTTCAGACGTGTGCTCTTCCGATCTNNNNACCTCTCTC CTCCAGCTGAG
HEK site no10	FW	CTTTCCCTACACGACGCTCTTCCGATCTNNNNTCCACCTCC CCACTTCTCTT
	RV	GGAGTTCAGACGTGTGCTCTTCCGATCTNNNNGGTGAAATG AGCAAGGCACA
HEK site no11	FW	CTTTCCCTACACGACGCTCTTCCGATCTNNNNCCCTAAACC ACCTGCAGAGG
	RV	GGAGTTCAGACGTGTGCTCTTCCGATCTNNNNCAGCCCCA GCCACATTCTAT
HEK site no14	FW	CTTTCCCTACACGACGCTCTTCCGATCTNNNNGAACCTGAA GCCTTTCCCCA
	RV	GGAGTTCAGACGTGTGCTCTTCCGATCTNNNNAACCTGTGT GACACTTGGCA
HEK site no16	FW	CTTTCCCTACACGACGCTCTTCCGATCTNNNNGGGAGGTG GAGAGAGGATGT
	RV	GGAGTTCAGACGTGTGCTCTTCCGATCTNNNNTCCTGAGGT CTAGGAACCCG
HEK site no18	FW	CTTTCCCTACACGACGCTCTTCCGATCTNNNNGCATTACCT GGGAGCCTGTT
	RV	GGAGTTCAGACGTGTGCTCTTCCGATCTNNNNAACTTCAGC GGGCATCAGAA

4.3 Oligonucleotides used for sgRNA cloning

**Oligonucleotide used for cloning of sgRNA:**		**Sequence (5'-3')**
DOCK3 no2	FW	caccgTAAGACTGAACAAGAATGGT
	RV	aaacACCATTCTTGTTCAGTCTTAC
TARDBP no2	FW	caccgCGGGAGTTCTTCTCTCAGTA
	RV	aaacTACTGAGAGAAGAACTCCCGC
ZNF212 no1	FW	caccgTGCACCTGGCATCAACAcgg
	RV	aaacCCGTGTTGATGCCAGGTGCAC
NEK1 no2	FW	caccgTGGCTCTCTCTACATAGTAA
	RV	aaacTTACTATGTAGAGAGAGCCAC
RSF1 no1	FW	caccgTTCATTCCCCCTGTCACACG
	RV	aaacCGTGTGACAGGGGGAATGAAC
RSF1 no2	FW	caccgACCCATTAAAGTTGAGGTGA
	RV	aaacTCACCTCAACTTTAATGGGTC
FANCF no3	FW	caccgAGCGGCGGCTGCACAACCAG
	RV	aaacCTGGTTGTGCAGCCGCCGCTC
FANCF no5	FW	caccgAGGCCCGGCGCACGGTGGCG
	RV	aaacCGCCACCGTGCGCCGGGCCTC
EMX1 no1	FW	caccgGAGTCCGAGCAGAAGAAGAA
	RV	aaacTTCTTCTTCTGCTCGGACTCC

EMX1 no2	FW	caccgAGATTTATGCAAACGGGTTG
	RV	aaacCAACCCGTTTGCATAAATCTC
HEK site no1	FW	caccgGAACACAAAGCATAGACTGC
	RV	aaacGCAGTCTATGCTTTGTGTTCC
HEK site no2	FW	caccgGAGTATGAGGCATAGACTGC
	RV	aaacGCAGTCTATGCCTCATACTCC
HEK site no3	FW	caccgGTCAAGAAAGCAGAGACTGC
	RV	aaacGCAGTCTCTGCTTTCTTGACC
HEK site no8	FW	caccgGTAAACAAAGCATAGACTGA
	RV	aaacTCAGTCTATGCTTTGTTTACC
HEK site no9	FW	caccgGAAGACCAAGGATAGACTGC
	RV	aaacGCAGTCTATCCTTGGTCTTCC
HEK site no10	FW	caccgGAACATAAAGAATAGAATGA
	RV	aaacTCATTCTATTCTTTATGTTCC
HEK site no11	FW	caccgGGACAGGCAGCATAGACTGT
	RV	aaacACAGTCTATGCTGCCTGTCCC
HEK site no14	FW	caccgGGCTAAAGACCATAGACTGT
	RV	aaacACAGTCTATGGTCTTTAGCCC
HEK site no16	FW	caccgGGGAATAAATCATAGAATCC
	RV	aaacGGATTCTATGATTTATTCCCC
HEK site no18	FW	caccgACACACACACTTAGAATCTG
	RV	aaacCAGATTCTAAGTGTGTGTGTC

4.4 Sequences of SpCas9-base-editor plasmids used in this study

(a) CMV-ABEmax-P2A-GFP (Addgene no.112101)

NLS
Linker
TadA-TadA*
Nickase SpCas9(D10A)
P2A-GFP

ATGAAACGGACAGCCGACGGAAGCGAGTTCGAGTCACCAAAGAAGAAGCGGA
AAGTC_TCTGAAGTCGAGTTTAGCCACGAGTATTGGATGAGGCACGCACTGACC
CTGGCAAAGCGAGCATGGGATGAAAGAGAAGTCCCCGTGGGCGCCGTGCTGG
TGCACAACAATAGAGTGATCGGAGAGGGATGGAACAGGCCAATCGGCCGCCA
CGACCCTACCGCACACGCAGAGATCATGGCACTGAGGCAGGGAGGCCTGGTC
ATGCAGAATTACCGCCTGATCGATGCCACCCTGTATGTGACACTGGAGCCATG
CGTGATGTGCGCAGGAGCAATGATCCACAGCAGGATCGGAAGAGTGGTGTTCG
GAGCACGGGACGCCAAGACCGGCGCAGCAGGCTCCCTGATGGATGTGCTGCA
CCACCCCGGCATGAACCACCGGGTGGAGATCACAGAGGGAATCCTGGCAGAC

GAGTGCGCCGCCCTGCTGAGCGATTTCTTTAGAATGCGGAGACAGGAGATCAA
GGCCCAGAAGAAGGCACAGAGCTCCACCGACTCTGGAGGATCTAGCGGAGGA
TCCTCTGGAAGCGAGACACCAGGCACAAGCGAGTCCGCCACACCAGAGAGCT
CCGGCGGCTCCTCCGGAGGATCCTCTGAGGTGGAGTTTTCCCACGAGTACTGG
ATGAGACATGCCCTGACCCTGGCCAAGAGGGCACGCGATGAGAGGGAGGTGC
CTGTGGGAGCCGTGCTGGTGCTGAACAATAGAGTGATCGGCGAGGGCTGGAA
CAGAGCCATCGGCCTGCACGACCCAACAGCCCATGCCGAAATTATGGCCCTGA
GACAGGGCGGCCTGGTCATGCAGAACTACAGACTGATTGACGCCACCCTGTAC
GTGACATTCGAGCCTTGCGTGATGTGCGCCGGCGCCATGATCCACTCTAGGAT
CGGCCGCGTGGTGTTTGGCGTGAGGAACGCAAAAACCGGCGCCGCAGGCTCC
CTGATGGACGTGCTGCACTACCCCGGCATGAATCACCGCGTCGAAATTACCGA
GGGAATCCTGGCAGATGAATGTGCCGCCCTGCTGTGCTATTTCTTTCGGATGC
CTAGACAGGTGTTCAATGCTCAGAAGAAGGCCCAGAGCTCCACCGAC_TCCGG
AGGATCTAGCGGAGGCTCCTCTGGCTCTGAGACACCTGGCACAAGCGAGAGC
GCAACACCTGAAAGCAGCGGGGGCAGCAGCGGGGGGTCA_GACAAGAAGTAC
AGCATCGGCCTGGCCATCGGCACCAACTCTGTGGGCTGGGCCGTGATCACCG
ACGAGTACAAGGTGCCCAGCAAGAAATTCAAGGTGCTGGGCAACACCGACCGG
CACAGCATCAAGAAGAACCTGATCGGAGCCCTGCTGTTCGACAGCGGCGAAAC
AGCCGAGGCCACCCGGCTGAAGAGAACCGCCAGAAGAAGATACACCAGACGG
AAGAACCGGATCTGCTATCTGCAAGAGATCTTCAGCAACGAGATGGCCAAGGT
GGACGACAGCTTCTTCCACAGACTGGAAGAGTCCTTCCTGGTGGAAGAGGATA
AGAAGCACGAGCGGCACCCCATCTTCGGCAACATCGTGGACGAGGTGGCCTA
CCACGAGAAGTACCCCACCATCTACCACCTGAGAAAGAAACTGGTGGACAGCA
CCGACAAGGCCGACCTGCGGCTGATCTATCTGGCCCTGGCCCACATGATCAAG
TTCCGGGGCCACTTCCTGATCGAGGGCGACCTGAACCCCGACAACAGCGACG
TGGACAAGCTGTTCATCCAGCTGGTGCAGACCTACAACCAGCTGTTCGAGGAA
AACCCCATCAACGCCAGCGGCGTGGACGCCAAGGCCATCCTGTCTGCCAGACT
GAGCAAGAGCAGACGGCTGGAAAATCTGATCGCCCAGCTGCCCGGCGAGAAG
AAGAATGGCCTGTTCGGAAACCTGATTGCCCTGAGCCTGGGCCTGACCCCCAA
CTTCAAGAGCAACTTCGACCTGGCCGAGGATGCCAAACTGCAGCTGAGCAAGG
ACACCTACGACGACGACCTGGACAACCTGCTGGCCCAGATCGGCGACCAGTAC
GCCGACCTGTTTCTGGCCGCCAAGAACCTGTCCGACGCCATCCTGCTGAGCGA
CATCCTGAGAGTGAACACCGAGATCACCAAGGCCCCCCTGAGCGCCTCTATGA
TCAAGAGATACGACGAGCACCACCAGGACCTGACCCTGCTGAAAGCTCTCGTG
CGGCAGCAGCTGCCTGAGAAGTACAAAGAGATTTTCTTCGACCAGAGCAAGAA
CGGCTACGCCGGCTACATTGACGGCGGAGCCAGCCAGGAAGAGTTCTACAAG
TTCATCAAGCCCATCCTGGAAAAGATGGACGGCACCGAGGAACTGCTCGTGAA
GCTGAACAGAGAGGACCTGCTGCGGAAGCAGCGGACCTTCGACAACGGCAGC
ATCCCCCACCAGATCCACCTGGGAGAGCTGCACGCCATTCTGCGGCGGCAGG
AAGATTTTTACCCATTCCTGAAGGACAACCGGGAAAAGATCGAGAAGATCCTGA
CCTTCCGCATCCCCTACTACGTGGGCCCTCTGGCCAGGGGAAACAGCAGATTC
GCCTGGATGACCAGAAAGAGCGAGGAAACCATCACCCCCTGGAACTTCGAGGA
AGTGGTGGACAAGGGCGCTTCCGCCCAGAGCTTCATCGAGCGGATGACCAACT
TCGATAAGAACCTGCCCAACGAGAAGGTGCTGCCCAAGCACAGCCTGCTGTAC
GAGTACTTCACCGTGTATAACGAGCTGACCAAAGTGAAATACGTGACCGAGGG
AATGAGAAAGCCCGCCTTCCTGAGCGGCGAGCAGAAAAAGGCCATCGTGGAC
CTGCTGTTCAAGACCAACCGGAAAGTGACCGTGAAGCAGCTGAAAGAGGACTA
CTTCAAGAAAATCGAGTGCTTCGACTCCGTGGAAATCTCCGGCGTGGAAGATC

GGTTCAACGCCTCCCTGGGCACATACCACGATCTGCTGAAAATTATCAAGGACA
AGGACTTCCTGGACAATGAGGAAAACGAGGACATTCTGGAAGATATCGTGCTG
ACCCTGACACTGTTTGAGGACAGAGAGATGATCGAGGAACGGCTGAAAACCTA
TGCCCACCTGTTCGACGACAAAGTGATGAAGCAGCTGAAGCGGCGGAGATACA
CCGGCTGGGGCAGGCTGAGCCGGAAGCTGATCAACGGCATCCGGGACAAGCA
GTCCGGCAAGACAATCCTGGATTTCCTGAAGTCCGACGGCTTCGCCAACAGAA
ACTTCATGCAGCTGATCCACGACGACAGCCTGACCTTTAAAGAGGACATCCAGA
AAGCCCAGGTGTCCGGCCAGGGCGATAGCCTGCACGAGCACATTGCCAATCT
GGCCGGCAGCCCCGCCATTAAGAAGGGCATCCTGCAGACAGTGAAGGTGGTG
GACGAGCTCGTGAAAGTGATGGGCCGGCACAAGCCCGAGAACATCGTGATCG
AAATGGCCAGAGAGAACCAGACCACCCAGAAGGGACAGAAGAACAGCCGCGA
GAGAATGAAGCGGATCGAAGAGGGCATCAAAGAGCTGGGCAGCCAGATCCTG
AAAGAACACCCCGTGGAAAACACCCAGCTGCAGAACGAGAAGCTGTACCTGTA
CTACCTGCAGAATGGGCGGGATATGTACGTGGACCAGGAACTGGACATCAACC
GGCTGTCCGACTACGATGTGGACCATATCGTGCCTCAGAGCTTTCTGAAGGAC
GACTCCATCGACAACAAGGTGCTGACCAGAAGCGACAAGAACCGGGGCAAGA
GCGACAACGTGCCCTCCGAAGAGGTCGTGAAGAAGATGAAGAACTACTGGCG
GCAGCTGCTGAACGCCAAGCTGATTACCCAGAGAAAGTTCGACAATCTGACCA
AGGCCGAGAGAGGCGGCCTGAGCGAACTGGATAAGGCCGGCTTCATCAAGAG
ACAGCTGGTGGAAACCCGGCAGATCACAAAGCACGTGGCACAGATCCTGGACT
CCCGGATGAACACTAAGTACGACGAGAATGACAAGCTGATCCGGGAAGTGAAA
GTGATCACCCTGAAGTCCAAGCTGGTGTCCGATTTCCGGAAGGATTTCCAGTTT
TACAAAGTGCGCGAGATCAACAACTACCACCACGCCCACGACGCCTACCTGAA
CGCCGTCGTGGGAACCGCCCTGATCAAAAAGTACCCTAAGCTGGAAAGCGAGT
TCGTGTACGGCGACTACAAGGTGTACGACGTGCGGAAGATGATCGCCAAGAGC
GAGCAGGAAATCGGCAAGGCTACCGCCAAGTACTTCTTCTACAGCAACATCAT
GAACTTTTTCAAGACCGAGATTACCCTGGCCAACGGCGAGATCCGGAAGCGGC
CTCTGATCGAGACAAACGGCGAAACCGGGGAGATCGTGTGGGATAAGGGCCG
GGATTTTGCCACCGTGCGGAAAGTGCTGAGCATGCCCCAAGTGAATATCGTGA
AAAAGACCGAGGTGCAGACAGGCGGCTTCAGCAAAGAGTCTATCCTGCCCAAG
AGGAACAGCGATAAGCTGATCGCCAGAAAGAAGGACTGGGACCCTAAGAAGTA
CGGCGGCTTCGACAGCCCCACCGTGGCCTATTCTGTGCTGGTGGTGGCCAAA
GTGGAAAAGGGCAAGTCCAAGAAACTGAAGAGTGTGAAAGAGCTGCTGGGGAT
CACCATCATGGAAAGAAGCAGCTTCGAGAAGAATCCCATCGACTTTCTGGAAGC
CAAGGGCTACAAAGAAGTGAAAAAGGACCTGATCATCAAGCTGCCTAAGTACTC
CCTGTTCGAGCTGGAAAACGGCCGGAAGAGAATGCTGGCCTCTGCCGGCGAA
CTGCAGAAGGGAAACGAACTGGCCCTGCCCTCCAAATATGTGAACTTCCTGTA
CCTGGCCAGCCACTATGAGAAGCTGAAGGGCTCCCCCGAGGATAATGAGCAGA
AACAGCTGTTTGTGGAACAGCACAAGCACTACCTGGACGAGATCATCGAGCAG
ATCAGCGAGTTCTCCAAGAGAGTGATCCTGGCCGACGCTAATCTGGACAAAGT
GCTGTCCGCCTACAACAAGCACCGGGATAAGCCCATCAGAGAGCAGGCCGAG
AATATCATCCACCTGTTTACCCTGACCAATCTGGGAGCCCCTGCCGCCTTCAAG
TACTTTGACACCACCATCGACCGGAAGAGGTACACCAGCACCAAAGAGGTGCT
GGACGCCACCCTGATCCACCAGAGCATCACCGGCCTGTACGAGACACGGATC
GACCTGTCTCAGCTGGGAGGTGAC_TCTGGCGGCTCAA_AAAGAACCGCCGAC
GGCAGCGAATTCGAGCCCAAGAAGAAGAGGAAAGTC_TCTGGTGGTTCTCCCA
AGAAGAAGAGGAAAGTCGGAAGCGGAGCTACTAACTTCAGCCTGCTGAAGCAG
GCTGGAGACGTGGAGGAGAACCCTGGACCTATGGTGAGCAAGGGCGAGGAGC

TGTTCACCGGGGTGGTGCCCATCCTGGTCGAGCTGGACGGCGACGTAAACGG
CCACAAGTTCAGCGTGTCCGGCGAGGGCGAGGGCGATGCCACCTACGGCAAG
CTGACCCTGAAGTTCATCTGCACCACCGGCAAGCTGCCCGTGCCCTGGCCCAC
CCTCGTGACCACCCTGACCTACGGCGTGCAGTGCTTCAGCCGCTACCCCGACC
ACATGAAGCAGCACGACTTCTTCAAGTCCGCCATGCCCGAAGGCTACGTCCAG
GAGCGCACCATCTTCTTCAAGGACGACGGCAACTACAAGACCCGCGCCGAGGT
GAAGTTCGAGGGCGACACCCTGGTGAACCGCATCGAGCTGAAGGGCATCGAC
TTCAAGGAGGACGGCAACATCCTGGGGCACAAGCTGGAGTACAACTACAACAG
CCACAACGTCTATATCATGGCCGACAAGCAGAAGAACGGCATCAAGGTGAACT
TCAAGATCCGCCACAACATCGAGGACGGCAGCGTGCAGCTCGCCGACCACTAC
CAGCAGAACACCCCCATCGGCGACGGCCCCGTGCTGCTGCCCGACAACCACT
ACCTGAGCACCCAGTCCGCCCTGAGCAAAGACCCCAACGAGAAGCGCGATCA
CATGGTCCTGCTGGAGTTCGTGACCGCCGCCGGGATCACTCTCGGCATGGAC
GAGCTGTACAAGTCTGGTGGTTCTCCCAAGAAGAAGAGGAAAGTCTAA

(b) CMV-BE4max-P2A-GFP (Addgene no. 112099)

NLS
Linker
Rat APOBEC1
Nickase SpCas9(D10A)
UGI
P2A-GFP

ATGAAACGGACAGCCGACGGAAGCGAGTTCGAGTCACCAAAGAAGAAGCGGA
AAGTC_TCCTCAGAGACTGGGCCTGTCGCCGTCGATCCAACCCTGCGCCGCCG
GATTGAACCTCACGAGTTTGAAGTGTTCTTTGACCCCCGGGAGCTGAGAAAGG
AGACATGCCTGCTGTACGAGATCAACTGGGGAGGCAGGCACTCCATCTGGAGG
CACACCTCTCAGAACACAAATAAGCACGTGGAGGTGAACTTCATCGAGAAGTTT
ACCACAGAGCGGTACTTCTGCCCCAATACCAGATGTAGCATCACATGGTTTCTG
AGCTGGTCCCCTTGCGGAGAGTGTAGCAGGGCCATCACCGAGTTCCTGTCCAG
ATATCCACACGTGACACTGTTTATCTACATCGCCAGGCTGTATCACCACGCAGA
CCCAAGGAATAGGCAGGGCCTGCGCGATCTGATCAGCTCCGGCGTGACCATC
CAGATCATGACAGAGCAGGAGTCCGGCTACTGCTGGCGGAACTTCGTGAATTA
TTCTCCTAGCAACGAGGCCCACTGGCCTAGGTACCCACACCTGTGGGTGCGCC
TGTACGTGCTGGAGCTGTATTGCATCATCCTGGGCCTGCCCCCTTGTCTGAATA
TCCTGCGGAGAAAGCAGCCCCAGCTGACCTTCTTTACAATCGCCCTGCAGTCTT
GTCACTATCAGAGGCTGCCACCCCACATCCTGTGGGCCACAGGCCTGAAG_TC
TGGAGGATCTAGCGGAGGATCCTCTGGCAGCGAGACACCAGGAACAAGCGAG
TCAGCAACACCAGAGAGCAGTGGCGGCAGCAGCGGCGGCAGC_GACAAGAAG
TACAGCATCGGCCTGGCCATCGGCACCAACTCTGTGGGCTGGGCCGTGATCAC
CGACGAGTACAAGGTGCCCAGCAAGAAATTCAAGGTGCTGGGCAACACCGACC
GGCACAGCATCAAGAAGAACCTGATCGGAGCCCTGCTGTTCGACAGCGGCGAA
ACAGCCGAGGCCACCCGGCTGAAGAGAACCGCCAGAAGAAGATACACCAGAC
GGAAGAACCGGATCTGCTATCTGCAAGAGATCTTCAGCAACGAGATGGCCAAG
GTGGACGACAGCTTCTTCCACAGACTGGAAGAGTCCTTCCTGGTGGAAGAGGA
TAAGAAGCACGAGCGGCACCCCATCTTCGGCAACATCGTGGACGAGGTGGCCT
ACCACGAGAAGTACCCCACCATCTACCACCTGAGAAAGAAACTGGTGGACAGC

ACCGACAAGGCCGACCTGCGGCTGATCTATCTGGCCCTGGCCCACATGATCAA
GTTCCGGGGCCACTTCCTGATCGAGGGCGACCTGAACCCCGACAACAGCGAC
GTGGACAAGCTGTTCATCCAGCTGGTGCAGACCTACAACCAGCTGTTCGAGGA
AAACCCCATCAACGCCAGCGGCGTGGACGCCAAGGCCATCCTGTCTGCCAGA
CTGAGCAAGAGCAGACGGCTGGAAAATCTGATCGCCCAGCTGCCCGGCGAGA
AGAAGAATGGCCTGTTCGGAAACCTGATTGCCCTGAGCCTGGGCCTGACCCCC
AACTTCAAGAGCAACTTCGACCTGGCCGAGGATGCCAAACTGCAGCTGAGCAA
GGACACCTACGACGACGACCTGGACAACCTGCTGGCCCAGATCGGCGACCAG
TACGCCGACCTGTTTCTGGCCGCCAAGAACCTGTCCGACGCCATCCTGCTGAG
CGACATCCTGAGAGTGAACACCGAGATCACCAAGGCCCCCCTGAGCGCCTCTA
TGATCAAGAGATACGACGAGCACCACCAGGACCTGACCCTGCTGAAAGCTCTC
GTGCGGCAGCAGCTGCCTGAGAAGTACAAAGAGATTTTCTTCGACCAGAGCAA
GAACGGCTACGCCGGCTACATTGACGGCGGAGCCAGCCAGGAAGAGTTCTAC
AAGTTCATCAAGCCCATCCTGGAAAAGATGGACGGCACCGAGGAACTGCTCGT
GAAGCTGAACAGAGAGGACCTGCTGCGGAAGCAGCGGACCTTCGACAACGGC
AGCATCCCCCACCAGATCCACCTGGGAGAGCTGCACGCCATTCTGCGGCGGC
AGGAAGATTTTTACCCATTCCTGAAGGACAACCGGGAAAAGATCGAGAAGATCC
TGACCTTCCGCATCCCCTACTACGTGGGCCCTCTGGCCAGGGGAAACAGCAGA
TTCGCCTGGATGACCAGAAAGAGCGAGGAAACCATCACCCCCTGGAACTTCGA
GGAAGTGGTGGACAAGGGCGCTTCCGCCCAGAGCTTCATCGAGCGGATGACC
AACTTCGATAAGAACCTGCCCAACGAGAAGGTGCTGCCCAAGCACAGCCTGCT
GTACGAGTACTTCACCGTGTATAACGAGCTGACCAAAGTGAAATACGTGACCGA
GGGAATGAGAAAGCCCGCCTTCCTGAGCGGCGAGCAGAAAAAGGCCATCGTG
GACCTGCTGTTCAAGACCAACCGGAAAGTGACCGTGAAGCAGCTGAAAGAGGA
CTACTTCAAGAAAATCGAGTGCTTCGACTCCGTGGAAATCTCCGGCGTGGAAG
ATCGGTTCAACGCCTCCCTGGGCACATACCACGATCTGCTGAAAATTATCAAGG
ACAAGGACTTCCTGGACAATGAGGAAAACGAGGACATTCTGGAAGATATCGTG
CTGACCCTGACACTGTTTGAGGACAGAGAGATGATCGAGGAACGGCTGAAAAC
CTATGCCCACCTGTTCGACGACAAAGTGATGAAGCAGCTGAAGCGGCGGAGAT
ACACCGGCTGGGGCAGGCTGAGCCGGAAGCTGATCAACGGCATCCGGGACAA
GCAGTCCGGCAAGACAATCCTGGATTTCCTGAAGTCCGACGGCTTCGCCAACA
GAAACTTCATGCAGCTGATCCACGACGACAGCCTGACCTTTAAAGAGGACATCC
AGAAAGCCCAGGTGTCCGGCCAGGGCGATAGCCTGCACGAGCACATTGCCAA
TCTGGCCGGCAGCCCCGCCATTAAGAAGGGCATCCTGCAGACAGTGAAGGTG
GTGGACGAGCTCGTGAAAGTGATGGGCCGGCACAAGCCCGAGAACATCGTGA
TCGAAATGGCCAGAGAGAACCAGACCACCCAGAAGGGACAGAAGAACAGCCG
CGAGAGAATGAAGCGGATCGAAGAGGGCATCAAAGAGCTGGGCAGCCAGATC
CTGAAAGAACACCCCGTGGAAAACACCCAGCTGCAGAACGAGAAGCTGTACCT
GTACTACCTGCAGAATGGGCGGGATATGTACGTGGACCAGGAACTGGACATCA
ACCGGCTGTCCGACTACGATGTGGACCATATCGTGCCTCAGAGCTTTCTGAAG
GACGACTCCATCGACAACAAGGTGCTGACCAGAAGCGACAAGAACCGGGGCA
AGAGCGACAACGTGCCCTCCGAAGAGGTCGTGAAGAAGATGAAGAACTACTGG
CGGCAGCTGCTGAACGCCAAGCTGATTACCCAGAGAAAGTTCGACAATCTGAC
CAAGGCCGAGAGAGGCGGCCTGAGCGAACTGGATAAGGCCGGCTTCATCAAG
AGACAGCTGGTGGAAACCCGGCAGATCACAAAGCACGTGGCACAGATCCTGGA
CTCCCGGATGAACACTAAGTACGACGAGAATGACAAGCTGATCCGGGAAGTGA
AAGTGATCACCCTGAAGTCCAAGCTGGTGTCCGATTTCCGGAAGGATTTCCAGT
TTTACAAAGTGCGCGAGATCAACAACTACCACCACGCCCACGACGCCTACCTG

AACGCCGTCGTGGGAACCGCCCTGATCAAAAAGTACCCTAAGCTGGAAAGCGA
GTTCGTGTACGGCGACTACAAGGTGTACGACGTGCGGAAGATGATCGCCAAGA
GCGAGCAGGAAATCGGCAAGGCTACCGCCAAGTACTTCTTCTACAGCAACATC
ATGAACTTTTTCAAGACCGAGATTACCCTGGCCAACGGCGAGATCCGGAAGCG
GCCTCTGATCGAGACAAACGGCGAAACCGGGGAGATCGTGTGGGATAAGGGC
CGGGATTTTGCCACCGTGCGGAAAGTGCTGAGCATGCCCCAAGTGAATATCGT
GAAAAAGACCGAGGTGCAGACAGGCGGCTTCAGCAAAGAGTCTATCCTGCCCA
AGAGGAACAGCGATAAGCTGATCGCCAGAAAGAAGGACTGGGACCCTAAGAAG
TACGGCGGCTTCGACAGCCCCACCGTGGCCTATTCTGTGCTGGTGGTGGCCAA
AGTGGAAAAGGGCAAGTCCAAGAAACTGAAGAGTGTGAAAGAGCTGCTGGGGA
TCACCATCATGGAAAGAAGCAGCTTCGAGAAGAATCCCATCGACTTTCTGGAAG
CCAAGGGCTACAAAGAAGTGAAAAAGGACCTGATCATCAAGCTGCCTAAGTACT
CCCTGTTCGAGCTGGAAAACGGCCGGAAGAGAATGCTGGCCTCTGCCGGCGA
ACTGCAGAAGGGAAACGAACTGGCCCTGCCCTCCAAATATGTGAACTTCCTGTA
CCTGGCCAGCCACTATGAGAAGCTGAAGGGCTCCCCCGAGGATAATGAGCAGA
AACAGCTGTTTGTGGAACAGCACAAGCACTACCTGGACGAGATCATCGAGCAG
ATCAGCGAGTTCTCCAAGAGAGTGATCCTGGCCGACGCTAATCTGGACAAAGT
GCTGTCCGCCTACAACAAGCACCGGGATAAGCCCATCAGAGAGCAGGCCGAG
AATATCATCCACCTGTTTACCCTGACCAATCTGGGAGCCCCTGCCGCCTTCAAG
TACTTTGACACCACCATCGACCGGAAGAGGTACACCAGCACCAAAGAGGTGCT
GGACGCCACCCTGATCCACCAGAGCATCACCGGCCTGTACGAGACACGGATC
GACCTGTCTCAGCTGGGAGGTGAC_AGCGGCGGGAGCGGCGGGAGCGGGGG
GAGC_ACTAATCTGAGCGACATCATTGAGAAGGAGACTGGGAAACAGCTGGTC
ATTCAGGAGTCCATCCTGATGCTGCCTGAGGAGGTGGAGGAAGTGATCGGCAA
CAAGCCAGAGTCTGACATCCTGGTGCACACCGCCTACGACGAGTCCACAGATG
AGAATGTGATGCTGCTGACCTCTGACGCCCCCGAGTATAAGCCTTGGGCCCTG
GTCATCCAGGATTCTAACGGCGAGAATAAGATCAAGATGCTG_AGCGGAGGAT
CCGGAGGATCTGGAGGCAGC_ACCAACCTGTCTGACATCATCGAGAAGGAGAC
AGGCAAGCAGCTGGTCATCCAGGAGAGCATCCTGATGCTGCCCGAAGAAGTCG
AAGAAGTGATCGGAAACAAGCCTGAGAGCGATATCCTGGTCCATACCGCCTAC
GACGAGAGTACCGACGAAAATGTGATGCTGCTGACATCCGACGCCCCAGAGTA
TAAGCCCTGGGCTCTGGTCATCCAGGATTCCAACGGAGAGAACAAAATCAAAAT
GCTG_TCTGGCGGCTCA_AAAAGAACCGCCGACGGCAGCGAATTCGAGCCCAA
GAAGAAGAGGAAAGTC_TCTGGTGGTTCTCCCAAGAAGAAGAGGAAAGTCGGA
AGCGGAGCTACTAACTTCAGCCTGCTGAAGCAGGCTGGAGACGTGGAGGAGA
ACCCTGGACCTATGGTGAGCAAGGGCGAGGAGCTGTTCACCGGGGTGGTGCC
CATCCTGGTCGAGCTGGACGGCGACGTAAACGGCCACAAGTTCAGCGTGTCC
GGCGAGGGCGAGGGCGATGCCACCTACGGCAAGCTGACCCTGAAGTTCATCT
GCACCACCGGCAAGCTGCCCGTGCCCTGGCCCACCCTCGTGACCACCCTGAC
CTACGGCGTGCAGTGCTTCAGCCGCTACCCCGACCACATGAAGCAGCACGACT
TCTTCAAGTCCGCCATGCCCGAAGGCTACGTCCAGGAGCGCACCATCTTCTTC
AAGGACGACGGCAACTACAAGACCCGCGCCGAGGTGAAGTTCGAGGGCGACA
CCCTGGTGAACCGCATCGAGCTGAAGGGCATCGACTTCAAGGAGGACGGCAA
CATCCTGGGGCACAAGCTGGAGTACAACTACAACAGCCACAACGTCTATATCAT
GGCCGACAAGCAGAAGAACGGCATCAAGGTGAACTTCAAGATCCGCCACAACA
TCGAGGACGGCAGCGTGCAGCTCGCCGACCACTACCAGCAGAACACCCCCAT
CGGCGACGGCCCCGTGCTGCTGCCCGACAACCACTACCTGAGCACCCAGTCC
GCCCTGAGCAAAGACCCCAACGAGAAGCGCGATCACATGGTCCTGCTGGAGTT

CGTGACCGCCGCCGGGATCACTCTCGGCATGGACGAGCTGTACAAGTCTGGT
GGTTCTCCCAAGAAGAAGAGGAAAGTCTAA

(c) CMV-ABE8e (Addgene no. 138489)

NLS
Linker
ecTadA(8e)
Nickase SpCas9(D10A)

ATGAAACGGACAGCCGACGGAAGCGAGTTCGAGTCACCAAAGAAGAAGCGGA
AAGTC_TCTGAGGTGGAGTTTTCCCACGAGTACTGGATGAGACATGCCCTGACC
CTGGCCAAGAGGGCACGGGATGAGAGGGAGGTGCCTGTGGGAGCCGTGCTG
GTGCTGAACAATAGAGTGATCGGCGAGGGCTGGAACAGAGCCATCGGCCTGC
ACGACCCAACAGCCCATGCCGAAATTATGGCCCTGAGACAGGGCGGCCTGGT
CATGCAGAACTACAGACTGATTGACGCCACCCTGTACGTGACATTCGAGCCTTG
CGTGATGTGCGCCGGCGCCATGATCCACTCTAGGATCGGCCGCGTGGTGTTTG
GCGTGAGGAACTCAAAAAGAGGCGCCGCAGGCTCCCTGATGAACGTGCTGAA
CTACCCCGGCATGAATCACCGCGTCGAAATTACCGAGGGAATCCTGGCAGATG
AATGTGCCGCCCTGCTGTGCGATTTCTATCGGATGCCTAGACAGGTGTTCAATG
CTCAGAAGAAGGCCCAGAGCTCCATCAAC_TCCGGAGGATCTAGCGGAGGCTC
CTCTGGCTCTGAGACACCTGGCACAAGCGAGAGCGCAACACCTGAAAGCAGC
GGGGGCAGCAGCGGGGGGTCA_GACAAGAAGTACAGCATCGGCCTGGCCATC
GGCACCAACTCTGTGGGCTGGGCCGTGATCACCGACGAGTACAAGGTGCCCA
GCAAGAAATTCAAGGTGCTGGGCAACACCGACCGGCACAGCATCAAGAAGAAC
CTGATCGGAGCCCTGCTGTTCGACAGCGGCGAAACAGCCGAGGCCACCCGGC
TGAAGAGAACCGCCAGAAGAAGATACACCAGACGGAAGAACCGGATCTGCTAT
CTGCAAGAGATCTTCAGCAACGAGATGGCCAAGGTGGACGACAGCTTCTTCCA
CAGACTGGAAGAGTCCTTCCTGGTGGAAGAGGATAAGAAGCACGAGCGGCAC
CCCATCTTCGGCAACATCGTGGACGAGGTGGCCTACCACGAGAAGTACCCCAC
CATCTACCACCTGAGAAAGAAACTGGTGGACAGCACCGACAAGGCCGACCTGC
GGCTGATCTATCTGGCCCTGGCCCACATGATCAAGTTCCGGGGCCACTTCCTG
ATCGAGGGCGACCTGAACCCCGACAACAGCGACGTGGACAAGCTGTTCATCCA
GCTGGTGCAGACCTACAACCAGCTGTTCGAGGAAAACCCCATCAACGCCAGCG
GCGTGGACGCCAAGGCCATCCTGTCTGCCAGACTGAGCAAGAGCAGACGGCT
GGAAAATCTGATCGCCCAGCTGCCCGGCGAGAAGAAGAATGGCCTGTTCGGAA
ACCTGATTGCCCTGAGCCTGGGCCTGACCCCCAACTTCAAGAGCAACTTCGAC
CTGGCCGAGGATGCCAAACTGCAGCTGAGCAAGGACACCTACGACGACGACC
TGGACAACCTGCTGGCCCAGATCGGCGACCAGTACGCCGACCTGTTTCTGGCC
GCCAAGAACCTGTCCGACGCCATCCTGCTGAGCGACATCCTGAGAGTGAACAC
CGAGATCACCAAGGCCCCCCTGAGCGCCTCTATGATCAAGAGATACGACGAGC
ACCACCAGGACCTGACCCTGCTGAAAGCTCTCGTGCGGCAGCAGCTGCCTGA
GAAGTACAAAGAGATTTTCTTCGACCAGAGCAAGAACGGCTACGCCGGCTACA
TTGACGGCGGAGCCAGCCAGGAAGAGTTCTACAAGTTCATCAAGCCCATCCTG
GAAAAGATGGACGGCACCGAGGAACTGCTCGTGAAGCTGAACAGAGAGGACC
TGCTGCGGAAGCAGCGGACCTTCGACAACGGCAGCATCCCCCACCAGATCCA
CCTGGGAGAGCTGCACGCCATTCTGCGGCGGCAGGAAGATTTTTACCCATTCC

TGAAGGACAACCGGGAAAAGATCGAGAAGATCCTGACCTTCCGCATCCCCTAC
TACGTGGGCCCTCTGGCCAGGGGAAACAGCAGATTCGCCTGGATGACCAGAAA
GAGCGAGGAAACCATCACCCCCTGGAACTTCGAGGAAGTGGTGGACAAGGGC
GCTTCCGCCCAGAGCTTCATCGAGCGGATGACCAACTTCGATAAGAACCTGCC
CAACGAGAAGGTGCTGCCCAAGCACAGCCTGCTGTACGAGTACTTCACCGTGT
ATAACGAGCTGACCAAAGTGAAATACGTGACCGAGGGAATGAGAAAGCCCGCC
TTCCTGAGCGGCGAGCAGAAAAAGGCCATCGTGGACCTGCTGTTCAAGACCAA
CCGGAAAGTGACCGTGAAGCAGCTGAAAGAGGACTACTTCAAGAAAATCGAGT
GCTTCGACTCCGTGGAAATCTCCGGCGTGGAAGATCGGTTCAACGCCTCCCTG
GGCACATACCACGATCTGCTGAAAATTATCAAGGACAAGGACTTCCTGGACAAT
GAGGAAAACGAGGACATTCTGGAAGATATCGTGCTGACCCTGACACTGTTTGA
GGACAGAGAGATGATCGAGGAACGGCTGAAAACCTATGCCCACCTGTTCGACG
ACAAAGTGATGAAGCAGCTGAAGCGGCGGAGATACACCGGCTGGGGCAGGCT
GAGCCGGAAGCTGATCAACGGCATCCGGGACAAGCAGTCCGGCAAGACAATC
CTGGATTTCCTGAAGTCCGACGGCTTCGCCAACAGAAACTTCATGCAGCTGATC
CACGACGACAGCCTGACCTTTAAAGAGGACATCCAGAAAGCCCAGGTGTCCGG
CCAGGGCGATAGCCTGCACGAGCACATTGCCAATCTGGCCGGCAGCCCCGCC
ATTAAGAAGGGCATCCTGCAGACAGTGAAGGTGGTGGACGAGCTCGTGAAAGT
GATGGGCCGGCACAAGCCCGAGAACATCGTGATCGAAATGGCCAGAGAGAAC
CAGACCACCCAGAAGGGACAGAAGAACAGCCGCGAGAGAATGAAGCGGATCG
AAGAGGGCATCAAAGAGCTGGGCAGCCAGATCCTGAAAGAACACCCCGTGGAA
AACACCCAGCTGCAGAACGAGAAGCTGTACCTGTACTACCTGCAGAATGGGCG
GGATATGTACGTGGACCAGGAACTGGACATCAACCGGCTGTCCGACTACGATG
TGGACCATATCGTGCCTCAGAGCTTTCTGAAGGACGACTCCATCGACAACAAG
GTGCTGACCAGAAGCGACAAGAACCGGGGCAAGAGCGACAACGTGCCCTCCG
AAGAGGTCGTGAAGAAGATGAAGAACTACTGGCGGCAGCTGCTGAACGCCAAG
CTGATTACCCAGAGAAAGTTCGACAATCTGACCAAGGCCGAGAGAGGCGGCCT
GAGCGAACTGGATAAGGCCGGCTTCATCAAGAGACAGCTGGTGGAAACCCGG
CAGATCACAAAGCACGTGGCACAGATCCTGGACTCCCGGATGAACACTAAGTA
CGACGAGAATGACAAGCTGATCCGGGAAGTGAAAGTGATCACCCTGAAGTCCA
AGCTGGTGTCCGATTTCCGGAAGGATTTCCAGTTTTACAAAGTGCGCGAGATCA
ACAACTACCACCACGCCCACGACGCCTACCTGAACGCCGTCGTGGGAACCGC
CCTGATCAAAAAGTACCCTAAGCTGGAAAGCGAGTTCGTGTACGGCGACTACA
AGGTGTACGACGTGCGGAAGATGATCGCCAAGAGCGAGCAGGAAATCGGCAA
GGCTACCGCCAAGTACTTCTTCTACAGCAACATCATGAACTTTTTCAAGACCGA
GATTACCCTGGCCAACGGCGAGATCCGGAAGCGGCCTCTGATCGAGACAAAC
GGCGAAACCGGGGAGATCGTGTGGGATAAGGGCCGGGATTTTGCCACCGTGC
GGAAAGTGCTGAGCATGCCCCAAGTGAATATCGTGAAAAAGACCGAGGTGCAG
ACAGGCGGCTTCAGCAAAGAGTCTATCCTGCCCAAGAGGAACAGCGATAAGCT
GATCGCCAGAAAGAAGGACTGGGACCCTAAGAAGTACGGCGGCTTCGACAGC
CCCACCGTGGCCTATTCTGTGCTGGTGGTGGCCAAAGTGGAAAAGGGCAAGTC
CAAGAAACTGAAGAGTGTGAAAGAGCTGCTGGGGATCACCATCATGGAAAGAA
GCAGCTTCGAGAAGAATCCCATCGACTTTCTGGAAGCCAAGGGCTACAAAGAA
GTGAAAAAGGACCTGATCATCAAGCTGCCTAAGTACTCCCTGTTCGAGCTGGAA
AACGGCCGGAAGAGAATGCTGGCCTCTGCCGGCGAACTGCAGAAGGGAAACG
AACTGGCCCTGCCCTCCAAATATGTGAACTTCCTGTACCTGGCCAGCCACTATG
AGAAGCTGAAGGGCTCCCCCGAGGATAATGAGCAGAAACAGCTGTTTGTGGAA
CAGCACAAGCACTACCTGGACGAGATCATCGAGCAGATCAGCGAGTTCTCCAA

GAGAGTGATCCTGGCCGACGCTAATCTGGACAAAGTGCTGTCCGCCTACAACA
AGCACCGGGATAAGCCCATCAGAGAGCAGGCCGAGAATATCATCCACCTGTTT
ACCCTGACCAATCTGGGAGCCCCTGCCGCCTTCAAGTACTTTGACACCACCAT
CGACCGGAAGAGGTACACCAGCACCAAAGAGGTGCTGGACGCCACCCTGATC
CACCAGAGCATCACCGGCCTGTACGAGACACGGATCGACCTGTCTCAGCTGGG
AGGTGAC_TCTGGCGGCTCAA_AAAGAACCGCCGACGGCAGCGAATTCGAGCC
CAAGAAGAAGAGGAAAGTCTAA

(d) CAG-nSpCas9-pmCDA1-P2A-GFP (Target-AID; Addgene no. 131300)

NLS
Linker
Nickase SpCas9(D10A)
3xFLAG
pmCDA1
UGI
P2A-EGFP

ATGGCACCGAAGAAGAAGCGTAAAGTCGGAATCCACGGAGTTCCTGCGGCA_A
TGGACAAGAAGTACTCCATTGGGCTCGCTATCGGCACAAACAGCGTCGGTTGG
GCCGTCATTACGGACGAGTACAAGGTGCCGAGCAAAAAATTCAAAGTTCTGGG
CAATACCGATCGCCACAGCATAAAGAAGAACCTCATTGGCGCCCTCCTGTTCGA
CTCCGGGGAGACGGCCGAAGCCACGCGGCTCAAAAGAACAGCACGGCGCAGA
TATACCCGCAGAAAGAATCGGATCTGCTACCTGCAGGAGATCTTTAGTAATGAG
ATGGCTAAGGTGGATGACTCTTTCTTCCATAGGCTGGAGGAGTCCTTTTTGGTG
GAGGAGGATAAAAAGCACGAGCGCCACCCAATCTTTGGCAATATCGTGGACGA
GGTGGCGTACCATGAAAAGTACCCAACCATATATCATCTGAGGAAGAAGCTTGT
AGACAGTACTGATAAGGCTGACTTGCGGTTGATCTATCTCGCGCTGGCGCATAT
GATCAAATTTCGGGGACACTTCCTCATCGAGGGGGACCTGAACCCAGACAACA
GCGATGTCGACAAACTCTTTATCCAACTGGTTCAGACTTACAATCAGCTTTTCGA
AGAGAACCCGATCAACGCATCCGGAGTTGACGCCAAAGCAATCCTGAGCGCTA
GGCTGTCCAAATCCCGGCGGCTCGAAAACCTCATCGCACAGCTCCCTGGGGA
GAAGAAGAACGGCCTGTTTGGTAATCTTATCGCCCTGTCACTCGGGCTGACCC
CCAACTTTAAATCTAACTTCGACCTGGCCGAAGATGCCAAGCTTCAACTGAGCA
AAGACACCTACGATGATGATCTCGACAATCTGCTGGCCCAGATCGGCGACCAG
TACGCAGACCTTTTTTTGGCGGCAAAGAACCTGTCAGACGCCATTCTGCTGAGT
GATATTCTGCGAGTGAACACGGAGATCACCAAAGCTCCGCTGAGCGCTAGTAT
GATCAAGCGCTATGATGAGCACCACCAAGACTTGACTTTGCTGAAGGCCCTTGT
CAGACAGCAACTGCCTGAGAAGTACAAGGAAATTTTCTTCGATCAGTCTAAAAA
TGGCTACGCCGGATACATTGACGGCGGAGCAAGCCAGGAGGAATTTTACAAAT
TTATTAAGCCCATCTTGGAAAAAATGGACGGCACCGAGGAGCTGCTGGTAAAG
CTTAACAGAGAAGATCTGTTGCGCAAACAGCGCACTTTCGACAATGGAAGCATC
CCCCACCAGATTCACCTGGGCGAACTGCACGCTATCCTCAGGCGGCAAGAGGA
TTTCTACCCCTTTTTGAAAGATAACAGGGAAAAGATTGAGAAAATCCTCACATTT
CGGATACCCTACTATGTAGGCCCCCTCGCCCGGGGAAATTCCAGATTCGCGTG
GATGACTCGCAAATCAGAAGAGACCATCACTCCCTGGAACTTCGAGGAAGTCG
TGGATAAGGGGGCCTCTGCCCAGTCCTTCATCGAAAGGATGACTAACTTTGATA
AAAATCTGCCTAACGAAAAGGTGCTTCCTAAACACTCTCTGCTGTACGAGTACT

TCACAGTTTATAACGAGCTCACCAAGGTCAAATACGTCACAGAAGGGATGAGAA
AGCCAGCATTCCTGTCTGGAGAGCAGAAGAAAGCTATCGTGGACCTCCTCTTC
AAGACGAACCGGAAAGTTACCGTGAAACAGCTCAAAGAAGACTATTTCAAAAAG
ATTGAATGTTTCGACTCTGTTGAAATCAGCGGAGTGGAGGATCGCTTCAACGCA
TCCCTGGGAACGTATCACGATCTCCTGAAAATCATTAAAGACAAGGACTTCCTG
GACAATGAGGAGAACGAGGACATTCTTGAGGACATTGTCCTCACCCTTACGTTG
TTTGAAGATAGGGAGATGATTGAAGAACGCTTGAAAACTTACGCTCATCTCTTC
GACGACAAAGTCATGAAACAGCTCAAGAGGCGCCGATATACAGGATGGGGGC
GGCTGTCAAGAAAACTGATCAATGGGATCCGAGACAAGCAGAGTGGAAAGACA
ATCCTGGATTTTCTTAAGTCCGATGGATTTGCCAACCGGAACTTCATGCAGTTG
ATCCATGATGACTCTCTCACCTTTAAGGAGGACATCCAGAAAGCACAAGTTTCT
GGCCAGGGGGACAGTCTTCACGAGCACATCGCTAATCTTGCAGGTAGCCCAGC
TATCAAAAAGGGAATACTGCAGACCGTTAAGGTCGTGGATGAACTCGTCAAAGT
AATGGGAAGGCATAAGCCCGAGAATATCGTTATCGAGATGGCCCGAGAGAACC
AAACTACCCAGAAGGGACAGAAGAACAGTAGGGAAAGGATGAAGAGGATTGAA
GAGGGTATAAAAGAACTGGGGTCCCAAATCCTTAAGGAACACCCAGTTGAAAAC
ACCCAGCTTCAGAATGAGAAGCTCTACCTGTACTACCTGCAGAACGGCAGGGA
CATGTACGTGGATCAGGAACTGGACATCAATCGGCTCTCCGACTACGACGTGG
ATCATATCGTGCCCCAGTCTTTTCTCAAAGATGATTCTATTGATAATAAAGTGTT
GACAAGATCCGATAAAAATAGAGGGAAGAGTGATAACGTCCCCTCAGAAGAAG
TTGTCAAGAAAATGAAAAATTATTGGCGGCAGCTGCTGAACGCCAAACTGATCA
CACAACGGAAGTTCGATAATCTGACTAAGGCTGAACGAGGTGGCCTGTCTGAG
TTGGATAAAGCCGGCTTCATCAAAAGGCAGCTTGTTGAGACACGCCAGATCAC
CAAGCACGTGGCCCAAATTCTCGATTCACGCATGAACACCAAGTACGATGAAAA
TGACAAACTGATTCGAGAGGTGAAAGTTATTACTCTGAAGTCTAAGCTGGTCTC
AGATTTCAGAAAGGACTTTCAGTTTTATAAGGTGAGAGAGATCAACAATTACCAC
CATGCGCATGATGCCTACCTGAATGCAGTGGTAGGCACTGCACTTATCAAAAAA
TATCCCAAGCTTGAATCTGAATTTGTTTACGGAGACTATAAAGTGTACGATGTTA
GGAAAATGATCGCAAAGTCTGAGCAGGAAATAGGCAAGGCCACCGCTAAGTAC
TTCTTTTACAGCAATATTATGAATTTTTTCAAGACCGAGATTACACTGGCCAATG
GAGAGATTCGGAAGCGACCACTTATCGAAACAAACGGAGAAACAGGAGAAATC
GTGTGGGACAAGGGTAGGGATTTCGCGACAGTCCGGAAGGTCCTGTCCATGC
CGCAGGTGAACATCGTTAAAAAGACCGAAGTACAGACCGGAGGCTTCTCCAAG
GAAAGTATCCTCCCGAAAAGGAACAGCGACAAGCTGATCGCACGCAAAAAAGA
TTGGGACCCCAAGAAATACGGCGGATTCGATTCTCCTACAGTCGCTTACAGTGT
ACTGGTTGTGGCCAAAGTGGAGAAAGGGAAGTCTAAAAAACTCAAAAGCGTCA
AGGAACTGCTGGGCATCACAATCATGGAGCGATCAAGCTTCGAAAAAAACCCC
ATCGACTTTCTCGAGGCGAAAGGATATAAAGAGGTCAAAAAAGACCTCATCATT
AAGCTTCCCAAGTACTCTCTCTTTGAGCTTGAAAACGGCCGGAAACGAATGCTC
GCTAGTGCGGGCGAGCTGCAGAAAGGTAACGAGCTGGCACTGCCCTCTAAATA
CGTTAATTTCTTGTATCTGGCCAGCCACTATGAAAAGCTCAAAGGGTCTCCCGA
AGATAATGAGCAGAAGCAGCTGTTCGTGGAACAACACAAACACTACCTTGATGA
GATCATCGAGCAAATAAGCGAATTCTCCAAAAGAGTGATCCTCGCCGACGCTAA
CCTCGATAAGGTGCTTTCTGCTTACAATAAGCACAGGGATAAGCCCATCAGGGA
GCAGGCAGAAAACATTATCCACTTGTTTACTCTGACCAACTTGGGCGCGCCTGC
AGCCTTCAAGTACTTCGACACCACCATAGACAGAAAGCGGTACACCTCTACAAA
GGAGGTCCTGGACGCCACACTGATTCATCAGTCAATTACGGGGCTCTATGAAA
CAAGAATCGACCTCTCTCAGCTCGGTGGAGAC_AGCAGGGCTGAC_CCCAAGA

AGAAGAGGAAGGTG_GGTGGAGGAGGTACCGGCGGTGGAGGCTCAGCAGAAT
ACGTACGAGCTCTGTTTGACTTCAATGGGAATGACGAGGAGGATCTCCCCTTTA
AGAAGGGCGATATTCTCCGCATCAGAGATAAGCCCGAAGAACAATGGTGGAAT
GCCGAGGATAGCGAAGGGAAAAGGGGCATGATTCTGGTGCCATATGTGGAGAA
ATATTCCGGT_GACTACAAAGACCATGATGGGGATTACAAAGACCACGACATCG
ACTACAAAGACGACGACGATAAA_TCAGGGATGACAGACGCCGAGTACGTGCG
CATTCATGAGAAACTGGATATTTACACCTTCAAGAAGCAGTTCTTCAACAACAAG
AAATCTGTGTCACACCGCTGCTACGTGCTGTTTGAGTTGAAGCGAAGGGGCGA
AAGAAGGGCTTGCTTTTGGGGCTATGCCGTCAACAAGCCCCAAAGTGGCACCG
AGAGAGGAATACACGCTGAGATATTCAGTATCCGAAAGGTGGAAGAGTATCTTC
GGGATAATCCT_GGGCAGTTTACGATCAACTGGTATTCCAGCTGGAGTCCTTGC
GCTGATTGTGCCGAGAAAATTCTGGAATGGTATAATCAGGAACTTCGGGGAAAC
GGGCACACATTGAAAATCTGGGCCTGCAAGCTGTACTACGAGAAGAATGCCCG
GAACCAGATAGGACTCTGGAATCTGAGGGACAATGGTGTAGGCCTGAACGTGA
TGGTTTCCGAGCACTATCAGTGTTGTCGGAAGATTTTCATCCAAAGCTCTCATAA
CCAGCTCAATGAAAACCGCTGGTTGGAGAAAACACTGAAACGTGCGGAGAAGT
GGAGATCCGAGCTGAGCATCATGATCCAGGTCAAGATTCTGCATACCACTAAGT
CTCCAGCCGTTGGT_CCCAAGAAGAAAAGAAAAGTC_GGTACC_ATGACCAACC
TTTCCGACATCATAGAGAAGGAAACAGGCAAACAGTTGGTCATCCAAGAGTCGA
TACTCATGCTTCCTGAAGAAGTTGAGGAGGTCATTGGGAATAAGCCGGAAAGT
GACATTCTCGTACACACTGCGTATGATGAGAGCACCGATGAGAACGTGATGCT
GCTCACGTCAGATGCCCCAGAGTACAAACCCTGGGCTCTGGTGATTCAGGACT
CTAATGGAGAGAACAAGATCAAGATGCTA_GGAGGCGGTGGAAGCGGCGCAAC
AAACTTCTCTCTGCTGAAACAAGCCGGAGATGTCGAAGAGAATCCTGGACCGAT
GGTGAGCAAGGGCGAGGAGCTGTTCACCGGGGTGGTGCCCATCCTGGTCGAG
CTGGACGGCGACGTAAACGGCCACAAGTTCAGCGTGTCCGGCGAGGGCGAGG
GCGATGCCACCTACGGCAAGCTGACCCTGAAGTTCATCTGCACCACCGGCAAG
CTGCCCGTGCCCTGGCCCACCCTCGTGACCACCCTGACCTACGGCGTGCAGT
GCTTCAGCCGCTACCCCGACCACATGAAGCAGCACGACTTCTTCAAGTCCGCC
ATGCCCGAAGGCTACGTCCAGGAGCGCACCATCTTCTTCAAGGACGACGGCAA
CTACAAGACCCGCGCCGAGGTGAAGTTCGAGGGCGACACCCTGGTGAACCGC
ATCGAGCTGAAGGGCATCGACTTCAAGGAGGACGGCAACATCCTGGGGCACAA
GCTGGAGTACAACTACAACAGCCACAACGTCTATATCATGGCCGACAAGCAGA
AGAACGGCATCAAGGTGAACTTCAAGATCCGCCACAACATCGAGGACGGCAGC
GTGCAGCTCGCCGACCACTACCAGCAGAACACCCCCATCGGCGACGGCCCCG
TGCTGCTGCCCGACAACCACTACCTGAGCACCCAGTCCGCCCTGAGCAAAGAC
CCCAACGAGAAGCGCGATCACATGGTCCTGCTGGAGTTCGTGACCGCCGCCG
GGATCACTCTCGGCATGGACGAGCTGTACAAGTGA

5 References

[1] M. F. Richter, K. T. Zhao, E. Eton, A. Lapinaite, G. A. Newby, B. W. Thuronyi, C. Wilson, L. W. Koblan, J. Zeng, D. E. Bauer, J. A. Doudna, and D. R. Liu, "Phage-assisted evolution of an adenine base editor with improved Cas domain compatibility and activity," *Nature Biotechnology*, pp. 1–9, mar 2020. [Online]. Available: https://www.nature.com/articles/s41587-020-0453-z

[2] A. C. Komor, K. T. Zhao, M. S. Packer, N. M. Gaudelli, A. L. Waterbury, L. W. Koblan, Y. B. Kim, A. H. Badran, and D. R. Liu, "Improved base excision repair inhibition and bacteriophage Mu Gam protein yields C:G-to-T:A base editors with higher efficiency and product purity," vol. 3, no. 8, p. eaao4774, aug 2017. [Online]. Available: https://advances.sciencemag.org/content/3/8/eaao4774https://advances.sciencemag.org/content/3/8/eaao4774.abstract

[3] M. Song, H. K. Kim, S. Lee, Y. Kim, S. Y. Seo, J. Park, J. W. Choi, H. Jang, J. H. Shin, S. Min, Z. Quan, J. H. Kim, H. C. Kang, S. Yoon, and H. H. Kim, "Sequence-specific prediction of the efficiencies of adenine and cytosine base editors," *Nature Biotechnology*, vol. 38, no. 9, pp. 1037–1043, sep 2020.

[4] M. Arbab, M. W. Shen, B. Mok, C. Wilson, Ż. Matuszek, C. A. Cassa, and D. R. Liu, "Determinants of Base Editing Outcomes from Target Library Analysis and Machine Learning," *Cell*, jun 2020.

[5] A. Vaswani, N. Shazeer, N. Parmar, J. Uszkoreit, L. Jones, A. N. Gomez, L. Kaiser, and I. Polosukhin, "Attention Is All You Need," jun 2017. [Online]. Available: http://arxiv.org/abs/1706.03762

[6] A. Paszke, S. Gross, S. Chintala, G. Chanan, E. Yang, Z. Devito, Z. Lin, A. Desmaison, L. Antiga, and A. Lerer, "Automatic differentiation in pytorch," 2017.

[7] K. He, X. Zhang, S. Ren, and J. Sun, "Deep residual learning for image recognition," in *Proceedings of the IEEE Computer Society Conference on Computer Vision and Pattern Recognition*, vol. 2016-December. IEEE Computer Society, dec 2016, pp. 770–778.

[8] J. L. Ba, J. R. Kiros, and G. E. Hinton, "Layer Normalization," jul 2016. [Online]. Available: http://arxiv.org/abs/1607.06450

[9] J. Bergstra and Y. Bengio, "Random Search for HyperParameter Optimization," *Journal of Machine Learning Research*, 2012.
