# Supplementary material for: Predicting base editing outcomes with an attention-based deep learning algorithm trained on high-throughput target library screens
Source: Nat Commun. 2021 Aug 25;12:5114. doi: 10.1038/s41467-021-25375-z (PMC8387386; doi:10.1038/s41467-021-25375-z)
Supplement: Supplementary file 6 — Description of Additional Supplementary Files [file 41467_2021_25375_MOESM6_ESM.pdf]

Title: Supplementary Data 1:
Description: Per-base editing efficiencies and editing outcome proportions derived from high-throughput base editing screens. This file provides the basis for training and testing the BE-DICT models for the respective base editor.

Title: Supplementary Data 2:
Description: Editing rates measured at endogenous genomic loci upon base editing.

Title: Supplementary Data 3:
Description: Datasets for the performance evaluation of BE-DICT, BE-Hive and DeepBaseEditor. This file contains datasets from this study as well as datasets derived from the respective publications accompanying the publicly available models (Arbab et al., 2020; Song et al., 2020).
