# Supplementary material for: Predicting base editing outcomes with an attention-based deep learning algorithm trained on high-throughput target library screens
Source: Nat Commun. 2021 Aug 25;12:5114. doi: 10.1038/s41467-021-25375-z (PMC8387386; doi:10.1038/s41467-021-25375-z)
Supplement: Supplementary file 7 — Reporting Summary [file 41467_2021_25375_MOESM7_ESM.pdf]

nature research

Corresponding author(s): Prof. Gerald Schwank, Prof. Michael Krauthammer

Last updated by author(s): Jul 25, 2021

Reporting Summary

Nature Research wishes to improve the reproducibility of the work that we publish. This form provides structure for consistency and transparency in reporting. For further information on Nature Research policies, see our Editorial Policies and the Editorial Policy Checklist.

Statistics

For all statistical analyses, confirm that the following items are present in the figure legend, table legend, main text, or Methods section.

n/a	Confirmed	
☐	☒	The exact sample size (*n*) for each experimental group/condition, given as a discrete number and unit of measurement
☐	☒	A statement on whether measurements were taken from distinct samples or whether the same sample was measured repeatedly
☒	☐	The statistical test(s) used AND whether they are one- or two-sided<br>*Only common tests should be described solely by name; describe more complex techniques in the Methods section.*
☐	☒	A description of all covariates tested
☐	☒	A description of any assumptions or corrections, such as tests of normality and adjustment for multiple comparisons
☐	☒	A full description of the statistical parameters including central tendency (e.g. means) or other basic estimates (e.g. regression coefficient) AND variation (e.g. standard deviation) or associated estimates of uncertainty (e.g. confidence intervals)
☒	☐	For null hypothesis testing, the test statistic (e.g. *F*, *t*, *r*) with confidence intervals, effect sizes, degrees of freedom and *P* value noted<br>*Give P values as exact values whenever suitable.*
☒	☐	For Bayesian analysis, information on the choice of priors and Markov chain Monte Carlo settings
☒	☐	For hierarchical and complex designs, identification of the appropriate level for tests and full reporting of outcomes
☐	☒	Estimates of effect sizes (e.g. Cohen's *d*, Pearson's *r*), indicating how they were calculated

Our web collection on statistics for biologists contains articles on many of the points above.

Software and code

Policy information about availability of computer code

Data collection	Illumina NovaSeq RTA3 software v1.3.1 and MiSeq Control Software (MCS) v4.0 were used for deep sequencing data collection.
Data analysis	Data analysis was performed using R (version 3.5.2), Python3 and Microsoft Excel (version, 16.29). Fastq reads obtained from deep sequencing were trimmed using Cutadapt v2.2. The trimmed reads were then mapped using bowtie2 v2.3.5.1. Source code for BE-DICT and custom Python scripts used for the base editing frequency and outcomes calculations are available on GitHub at https://github.com/uzh-dqbm-cmi/crispr and https://github.com/sharan-j/GenCountTable.

For manuscripts utilizing custom algorithms or software that are central to the research but not yet described in published literature, software must be made available to editors and reviewers. We strongly encourage code deposition in a community repository (e.g. GitHub). See the Nature Research guidelines for submitting code & software for further information.

Data

Policy information about availability of data

All manuscripts must include a data availability statement. This statement should provide the following information, where applicable:

- Accession codes, unique identifiers, or web links for publicly available datasets
- A list of figures that have associated raw data
- A description of any restrictions on data availability

We used publicly available SNPs dataset from ClinVar (version : clinvar_20190319_hg38). We have provided the data sets used in this study as Supplementary Data 1-3. DNA-sequencing data are deposited under accession number PRJNA735610 (NCBI Sequence Read Archive) (https://www.ncbi.nlm.nih.gov/bioproject/?term=PRJNA735610). We have made the source code for BE-DICT and the custom Python scripts used to train and evaluate the models available on GitHub at https://github.com/uzh-dqbm-cmi/crispr and https://github.com/sharan-j/GenCountTable. The web application for BE-DICT is available at www.be-dict.org.

Field-specific reporting

Please select the one below that is the best fit for your research. If you are not sure, read the appropriate sections before making your selection.

- [x] Life sciences
- [ ] Behavioural & social sciences
- [ ] Ecological, evolutionary & environmental sciences

For a reference copy of the document with all sections, see nature.com/documents/nr-reporting-summary-flat.pdf

Life sciences study design

All studies must disclose on these points even when the disclosure is negative.

Sample size	No statistical analysis was used to predetermine the sample size. Sample sizes were determined based on the results of other groups in the field who generate reproducible results with similar setups (see Shen and Arbab et al., 2018, nature, doi: 10.1038/s41586-018-0686-x; Kim et al., 2019 DOI: 10.1126/sciadv.aax9249).
Data exclusions	To increase the accuracy of the analysis for base editing frequency, deep sequencing data were filtered to exclude target sequences with total Deep sequencing read counts below 100. To predict base editing efficiencies and outcomes for the modeling and therapeutic correction of human disease-relevant mutations, we used publicly available SNPs dataset from ClinVar (version : clinvar_20190319_hg38). The SNPs from the ClinVar dataset were filtered according to the following steps: (a) All disease associated SNPs were accessed and restricted to pathogenic and monogenic filters (b) SNPs were further restricted to the possible base conversions targetable by ABEs (A-to-G) and CBEs (C-to-G) (c) Genomic region flanking the SNP genomic coordinates were extracted from UCSC server (link) (d) The sequences were then scanned presence of an NGG PAM 8-to-18 bases away from the target base. Only SNPs passing these filtering criteria were included in the study and were then appended to the list of aforementioned random sequences to form the final library. These criteria were not pre-established.
Replication	The base-editing of the target library was performed as experimental duplicates. All base-editing experiments on endogenous genomic loci was replicated at least 2 times. All attempts were successful. Machine learning experiments were performed five times (denoted by runs). All attempts were successful.
Randomization	This study is not a case-control cohort experiment in life science, randomization was thus not applied.
Blinding	The investigators were not blinded to group allocation. This study does not involve animals or human research participants.

Reporting for specific materials, systems and methods

We require information from authors about some types of materials, experimental systems and methods used in many studies. Here, indicate whether each material, system or method listed is relevant to your study. If you are not sure if a list item applies to your research, read the appropriate section before selecting a response.

Materials & experimental systems

n/a	Involved in the study	
☒	☐	Antibodies
☐	☒	Eukaryotic cell lines
☒	☐	Palaeontology and archaeology
☒	☐	Animals and other organisms
☒	☐	Human research participants
☒	☐	Clinical data
☒	☐	Dual use research of concern

Methods

n/a	Involved in the study	
☒	☐	ChIP-seq
☒	☐	Flow cytometry
☒	☐	MRI-based neuroimaging

Eukaryotic cell lines

Policy information about cell lines

Cell line source(s)	HEK293T cells; source: American Type Culture Collection (ATCC) no. CRL-3216
Authentication	STR profiling by ATCC
Mycoplasma contamination	All cell lines tested negative for Mycoplasma contamination.
Commonly misidentified lines (See ICLAC register)	HEK293T are not listed in the ICLAC register (version 9)
